# Muscle Tissue Damage Induced by the Venom of *Bothrops asper*: Identification of Early and Late Pathological Events through Proteomic Analysis

**DOI:** 10.1371/journal.pntd.0004599

**Published:** 2016-04-01

**Authors:** Cristina Herrera, Jéssica Kele A. Macêdo, Andrés Feoli, Teresa Escalante, Alexandra Rucavado, José María Gutiérrez, Jay W. Fox

**Affiliations:** 1 Facultad de Farmacia, Universidad de Costa Rica, San José, Costa Rica; 2 Instituto Clodomiro Picado, Facultad de Microbiología, Universidad de Costa Rica, San José, Costa Rica; 3 University of Virginia School of Medicine, Charlottesville, Virginia, United States of America; Liverpool School of Tropical Medicine, UNITED KINGDOM

## Abstract

The time-course of the pathological effects induced by the venom of the snake *Bothrops asper* in muscle tissue was investigated by a combination of histology, proteomic analysis of exudates collected in the vicinity of damaged muscle, and immunodetection of extracellular matrix proteins in exudates. Proteomic assay of exudates has become an excellent new methodological tool to detect key biomarkers of tissue alterations for a more integrative perspective of snake venom-induced pathology. The time-course analysis of the intracellular proteins showed an early presence of cytosolic and mitochondrial proteins in exudates, while cytoskeletal proteins increased later on. This underscores the rapid cytotoxic effect of venom, especially in muscle fibers, due to the action of myotoxic phospholipases A_2_, followed by the action of proteinases in the cytoskeleton of damaged muscle fibers. Similarly, the early presence of basement membrane (BM) and other extracellular matrix (ECM) proteins in exudates reflects the rapid microvascular damage and hemorrhage induced by snake venom metalloproteinases. The presence of fragments of type IV collagen and perlecan one hour after envenoming suggests that hydrolysis of these mechanically/structurally-relevant BM components plays a key role in the genesis of hemorrhage. On the other hand, the increment of some ECM proteins in the exudate at later time intervals is likely a consequence of the action of endogenous matrix metalloproteinases (MMPs) or of *de novo* synthesis of ECM proteins during tissue remodeling as part of the inflammatory reaction. Our results offer relevant insights for a more integrative and systematic understanding of the time-course dynamics of muscle tissue damage induced by *B*. *asper* venom and possibly other viperid venoms.

## Author Summary

The local pathology induced by viperid snakes is characterized by a complex of alterations as consequence of direct and indirect effects of the toxins present in the venom, as well as the host response to tissue damage, and constitutes a dynamic process of degenerative and reparative events. The pathogenesis of local effects induced by *Bothrops asper* venom has been studied by traditional methodologies. Recently, proteomic analysis of wound exudates collected in the vicinity of affected tissue has become a powerful tool to study the pathogenesis of local envenoming from a more integrative perspective. Thus, in the present study we analyzed the dynamics of the local effects induced by *B*. *asper* venom in the gastrocnemius muscle of mice through a proteomic and immunochemistry approach in order to identify biomarkers of tissue damage and repair during the course of envenoming. Our results showed an early presence of cytosolic and mitochondrial proteins in exudates as compared to cytoskeletal proteins, which reflect the rapid cytotoxic effect of venom, followed by the action of endogenous proteinases in the cytoskeleton of damaged muscle fibers later on in the course of envenoming. On the other hand, the early presence of extracellular matrix components and the increment of some of them in exudates, reflect the rapid microvascular damage and hemorrhage induced by the venom, followed by the action of endogenous matrix metalloproteinases (MMPs) during tissue remodeling as part of the inflammatory response. Overall our study allowed the identification of key biomarkers of tissue damage and repair as part of the pathological effects induced by *B*. *asper* venom in skeletal muscle, which offer relevant insights for a better understanding of the complex dynamics of local pathology induced by viperid snakebite envenoming.

## Introduction

The viperid snake *Bothrops asper* is responsible for most snakebite cases in Central America and some regions of Mexico and South America [1,2]. The local pathology induced by viperid snakes is characterized by edema, blistering, hemorrhage, lymphatic vessel damage, and necrosis of skin and muscle, some of which can be attributed to the degradation of extracellular matrix (ECM) [1,3]. Such alterations develop very rapidly after the bite, and in some cases can lead to permanent tissue damage, regardless of the application of antivenom treatment. Significant efforts have been undertaken over the last several decades to identify the toxins responsible for these effects, as well as to characterize the pathogenesis of these alterations [3–5]. Nevertheless, the complexity of this pathology demands further analyses into hitherto unknown aspects of tissue damage and the complex interplay between degenerative and early reparative events. As envenoming is a dynamic event, it is critical to investigate the process over time, which is the main focus of this study.

The pathogenesis of local effects induced by *B*. *asper* venom has been studied by traditional methodologies, such as histological and ultrastructural analyses, immunohistochemical methods, and quantification of particular components and tissue markers in tissue homogenates or fluids, as a consequence of the action of crude venom and purified toxins [3,6–12]. Despite significant advances in the study of local tissue damage with these approaches, subtle changes in key biomarkers of tissue damage and repair during the course of envenoming remain to be identified and characterized. Moreover, since the venom is a highly complex mixture of components functioning over time, relevant information related to synergistic action of toxins could be missed when working only with isolated toxins; therefore, studies with crude venom may better advance our understanding from a predominantly reductionist to a holistic view of these multifactorial time-dependent phenomena.

Recently, proteomic analysis of exudates collected around the affected tissue has become a new methodological tool to study the pathogenesis of local tissue damage induced by snake venom from a more integrative perspective [13–17]. This approach has been used to study the alterations caused by *B*. *asper* snake venom [15], and some of its toxins, such as a myotoxic phospholipase A_2_ (PLA_2_) and a hemorrhagic snake venom metalloproteinase (SVMP) [13,14,16]. Moreover, proteomic analysis has allowed the comparison between the action of different types of hemorrhagic and non-hemorrhagic SVMPs [13,16,17]. These studies have identified differences in the species and abundance of intracellular proteins, ECM components, and other proteins present in exudates, which offer new insights in the mechanism of action of these toxins, and in the tissue damage induced by the venom [13–16]. However, these studies have been carried only at early time periods in the course of envenoming and therefore provide only a narrow window within the whole scenario of local pathology.

In the present study we analyzed the time-course variation in the protein composition and abundance of wound exudates collected from mouse gastrocnemius muscle injected with *B*. *asper* snake venom utilizing a proteomic and immunochemistry approach, in conjunction with histological analysis of tissue alterations, with the aim of identifying biomarkers of tissue damage and tissue remodeling characteristic of early and late stages of envenoming. This approach allowed the identification of key differences in some intracellular proteins and ECM components over time, which underscores the rapid cytotoxic and hemorrhagic effect of venom, followed by the action of endogenous proteinases associated with tissue remodeling later on in the course of envenoming. These results offer relevant insights for a better understanding of the complex pathological phenomena of viperid snakebite envenoming.

## Methods

### Venom

*B*. *asper* venom was obtained from more than 40 adult specimens collected in the Pacific region of Costa Rica and maintained at the serpentarium of Instituto Clodomiro Picado. After collection, venoms were pooled, lyophilized, and stored at -20°C until used.

### Ethics statement

CD-1 mice with a body weight between 18 and 20 g were used for the *in vivo* studies. All the experimental protocols involving the use of animals were approved by the Institutional Committee for the Care and Use of Laboratory Animals (CICUA) of the University of Costa Rica (protocol approval number CICUA 025–15), and meet the International Guiding Principles for Biomedical Research Involving Animals (CIOMS).

### Histology

Groups of four CD-1 mice (18–20 g) were injected in the right gastrocnemius with 50 μg of *B*. *asper* venom, dissolved in 50 μL of 0.12 M NaCl, 0.04 M phosphate, pH 7.2 solution (PBS). Control mice were injected with PBS alone. After 1, 6 and 24 h of injection, mice were sacrificed by CO_2_ inhalation and samples of the injected muscles were resected and added to 10% formalin solution in PBS. After 48 h fixation, routine processing of tissues was performed, followed by embedding in paraffin. Sections of 5 μm thickness were obtained for each sample and stained with hematoxylin–eosin for light microscopic observation.

### Collection of wound exudates

Groups of five CD-1 mice (18–20 g) were injected in the right gastrocnemius with 50 μg of *B*. *asper* venom, dissolved in 50 μL of PBS. After 1, 6 and 24 h of injection, mice were sacrificed by CO_2_ inhalation, a 5 mm incision was made with a scalpel in the skin overlying the injected muscle, and a heparinized capillary tube was introduced under the skin to collect the wound exudate fluid. An approximate volume of 20–50 μL of exudate was collected from each mouse. Exudate samples were then pooled and lyophilized for further analysis.

### Quantification of creatine kinase (CK) activity in wound exudates

Wound exudates were collected as previously described and centrifuged at 5000 g for 3 min. The CK activity of supernatants was determined using a commercial kit (CK-Nac, Biocon Diagnostik, Germany). CK activity was expressed in International Units /L (IU/L).

### Proteomic analysis of wound exudates

Lyophilized wound exudate samples were resuspended in water and protein quantification was performed using micro BCA protein assay kit (Thermo Scientific). Twenty micrograms of protein was precipitated with acetone, resuspended in Laemmli buffer and separated in a 5–20% precast electrophoresis gel (Bio-Rad). The gel was stained with Coomassie Brilliant Blue and lanes were cut into 8 equal size slices. Gel slices were destained for 3 h and the proteins were reduced (10 mM dithiothreitol, DTT) and alkylated (50 mM iodoacetamide) at room temperature. Gel slices were then washed with 100 mM ammonium bicarbonate, dehydrated with acetonitrile and dried in a speed vac, followed by in-gel digestion with a solution of Promega modified trypsin (20 ng/μL) in 50 mM ammonium bicarbonate for 30 min on ice. Excess trypsin solution was removed and the digestion continued for 18 h at 37°C. The resulting tryptic peptides were extracted from gel slices with two 30 μL aliquots of a 50% acetonitrile/5% formic acid solution. These extracts were combined and dried to 15 μL for mass spectrometric (MS) analysis.

LC/MS/MS was performed using a Thermo Electron Orbitrap Velos ETD mass spectrometer system. Analytical columns were fabricated in-house by packing 0.5 cm of irregular C18 Beads (YMC Gel ODS-A, 12 nm, I-10-25 um) followed by 7.5 cm Jupiter 10 μm C18 packing material (Phenomenex, Torrance, CA) into 360 x 75 μm fused silica (Polymicro Technologies, Phoenix, AZ) behind a bottleneck. Samples were loaded directly onto these columns for the C18 analytical runs. 7 μL of the extract was injected, and the peptides were eluted from the column at 0.5 μL/min using an acetonitrile/0.1M acetic acid gradient (2–90% acetonitrile over 1 h). The instrument was set to Full MS (m/z 300–1600) resolution of 60,000 and programmed to acquire a cycle of one mass spectrum followed by collision-induced dissociation (CID) MS/MS performed in the ion trap on the twenty most abundant ions in a data-dependent mode. Dynamic exclusion was enabled with an exclusion list of 400 masses, duration of 60 seconds, and repeat count of 1. The electrospray voltage was set to 2.4 kV, and the capillary temperature was 265°C.

The data were analyzed by database searching using the Sequest search algorithm in Proteome Discoverer 1.4.1 against the Uniprot Mouse database from July 2014. Spectra generated were searched using carbamidomethylation on cysteine as a fixed modification, oxidation of methionine as a variable modification, 10 ppm parent tolerance and 1 Da fragment tolerance. All hits were required to be fully tryptic. The results were exported to Scaffold (version 4.3.2, Proteome Software Inc., Portland, OR) to validate MS/MS based peptide and protein identifications, and to visualize multiple datasets in a comprehensive manner. Confidence of protein identification in Scaffold is shown as ≥ 95% confidence (green coloration) and 80% to 94% confidence (yellow coloration). Relative quantization of proteins was performed by summing all data from the 8 gel slices for a particular sample in Scaffold and then displaying the Quantitative Value from the program. This number gives an average total of non-grouped spectral counts for a protein divided by the total non-grouping spectral counts for the 8 mass spectral runs from the gels slices from each lane (http://www.proteomesoftware.com/). The Quantitative Value allows a relative quantitative comparison between a specific protein from different samples and relative abundance between proteins for a particular exudate sample.

### Immunochemical detection of ECM proteins in wound exudates

For immunoblotting, 100 μg protein of each exudate sample were separated under reducing conditions on 4–15% Tris–HCl polyacrylamide gradient gels, and transferred to nitrocellulose membranes. Immunodetection was performed by incubating the membranes overnight at 4°C stirring with rabbit anti-type IV collagen polyclonal antibody at a dilution of 1:200 (Abcam ab19808), rabbit anti-nidogen 1 polyclonal antibody at a dilution of 1:500 (Abcam ab14511), rabbit anti-laminin polyclonal antibody at a dilution of 1:1,000 (Thermo PA1-32130), rabbit anti-type VI collagen polyclonal antibody at a dilution of 1:2,000 (Millipore AB7821), rabbit anti-type I collagen polyclonal antibody at a dilution of 1:1,000 (Abcam ab21286), or rabbit anti-fibronectin polyclonal antibody at a dilution of 1:3,000 (Abcam ab2413). The reaction was developed using anti-rabbit peroxidase antibody at a dilution of 1:5,000 (Jackson ImmunoResearch) and the chemiluminescent substrate Lumi-Light (Roche). Images were captured with the ChemiDoc XRS+ System (BioRad) and the analysis was performed with the ImageLab software (BioRad).

### Quantification of proteolytic activity of wound exudates

#### Gelatinase assay

Proteolytic activity on fluorescent gelatin of wound exudates was assessed using a commercial kit (EnzCheck protocol Gelatinase/Collagenase Assay Kit, Molecular Probes, Life Technologies) in order to determine whether active SVMPs are present in the wound exudates. Exudate samples were collected as described above at 1 h, 6 h and 24 h after intramuscular injection of 100 μg of *B*. *asper* venom. A venom dose of 100 μg, instead of 50 μg, was used in these experiments in order to increase the sensitivity of the assay for detection of proteinase activity. Exudate samples were pooled, centrifuged at 5,000 g for 3 min and kept at -70°C until the proteolytic assays were performed. 50 μL of each exudate sample were incubated with 20 μg of gelatin fluorescein conjugate substrate in a total volume of 200 μL, in a 96 well microplate, protected from light, at room temperature, for 24 h. Each sample was tested in triplicate and a reagent blank was included. Fluorescence intensity was measured in the BioTek Synergy HT microplate reader setting the absorption filter at 495 nm and the emission filter at 515 nm. In order to determine whether proteolytic activity detected in exudate samples is due to SVMP or endogenous proteases, neutralization and zymography assays were performed with the exudate samples.

#### Neutralization assay

Exudate samples collected at 1 h were incubated with polyclonal antibodies against the SVMP BaP1 for 20 min at 37°C prior to testing the exudate in the gelatinase activity assay described above. The antibody against BaP1 was obtained from the serum of rabbits immunized with BaP1; antibodies were purified by affinity chromatography. This antibody was used since BaP1 is the most abundant SVMP in the venom of adult *B*. *asper* snakes [18]. Previous studies showed that anti-BaP1 antibodies do not react with PIII SVMPs from *B*. *asper* venom and with MMPs [19,20]. Exudate samples collected at 1 h were selected for the neutralization assay since the highest proteolytic activity on fluorescent gelatin was observed at this time interval.

#### Zymography assay

Proteinase activity of exudate samples was visualized by gelatin zymography according to the method described by Herron et al. [21] and modified by Rucavado et al. [20]. Briefly, 10 μg protein of each exudate sample were separated on 7.5% SDS-polyacrylamide gel prepared with 0.50 mg/mL of Type A gelatin (Sigma Chemical Co., St Luis, MO). After electrophoretic run at 100 V, the gel was washed with 1% Triton X-100 for 30 min under agitation. Then, the gels were incubated with zymography buffer (50 mM Tris-HCl, 5 mM CaCl_2_, 2 g/L NaN_3_, pH 8.0) for 20 h at 37°C, stained for 2 h with Coomassie Blue R-250, and destained with water for 20 min.

## Results

### Pathological observations

**Fig 1:**
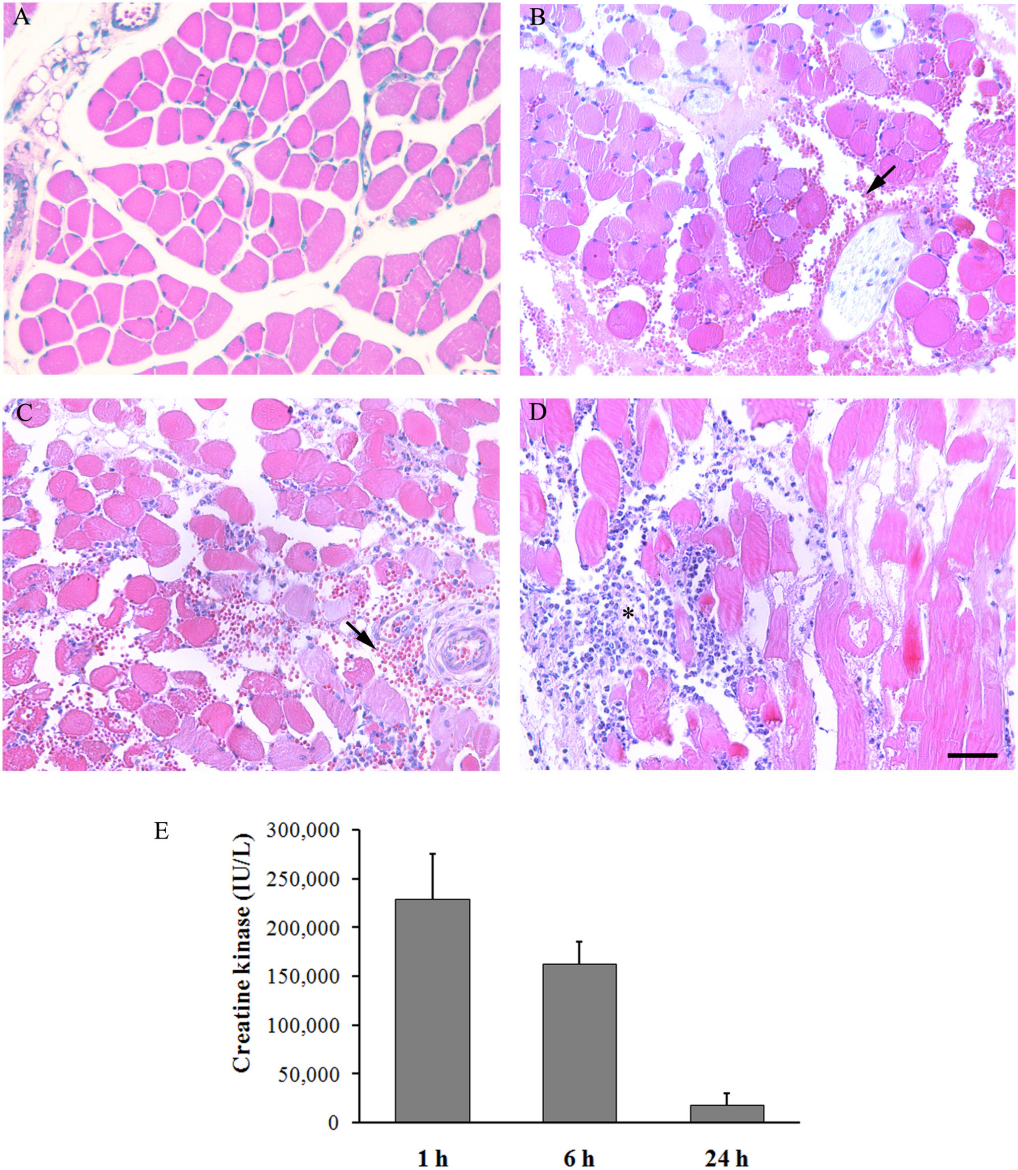
Histological analysis and myotoxicity induced by *B*. *asper* venom in mouse gastrocnemius muscle. Groups of four mice were injected in the gastrocnemius with 50 μg of *B*. *asper* venom. After 1, 6 and 24 h of injection, mice were sacrificed and samples of exudate and muscle tissues were collected for quantification of creatine kinase (CK) activity and histological analysis, respectively. Tissue samples were collected at 1 h (**B**), 6 h (**C**) and 24 h (**D**) after injection and processed for embedding in paraffin. Tissue injected with PBS (**A**) was used as control. Notice abundant erythrocytes (arrow) at 1 h and 6 h, and abundant infiltration of inflammatory cells at 24 h (asterisk). Hematoxylin–eosin staining. Bar represents 100 μm. (**E**) CK activity of exudate was quantified using a commercial kit (see Methods for details). Results are expressed as mean ± S.D (n = 4).

The pathological alterations induced by *B*. *asper* venom were studied on mouse gastrocnemius muscle tissue over a time period of 24 h. Tissue sections from control mice injected with PBS showed normal histological features of skeletal muscle tissue with transverse bundles of muscle fibers, surrounded by connective tissue and normal vascular and nerve structures (Fig 1A). Tissue sections from mice injected with *B*. *asper* venom showed intense hemorrhage at 1 h (Fig 1B) and 6 h (Fig 1C), evidenced by the presence of abundant erythrocytes in the interstitial space surrounding muscle fibers. After 24 h of injection of *B*. *asper* venom, the hemorrhage decreased since the amounts of extravascular erythrocytes was reduced as compared to previous time intervals (Fig 1D), consistent with previous observations [7].

Moreover, tissue sections from mice injected with venom revealed prominent necrosis of skeletal muscle fibers at the first hour interval (Fig 1B and 1C). After 24 h following venom injection, the bundles of muscle fibers appeared partially lost and disorganized with a hyaline appearance (Fig 1D). These pathological observations also agree with previous studies [7,11,12]. Additionally, an infiltration of inflammatory cells was observed in tissue sections, especially after 6 h and 24 h of venom injection with a marked increment at 24 h (Fig 1D). The predominant cell type was polymorphonuclear leukocytes, although a proportion of mononuclear cells, i.e. macrophages, were also observed at 24 h. These observations also agree with previous studies [9].

On the other hand, CK activity of exudate samples collected after injection of venom was 228,776 ± 47,137 IU/L at 1 h, 162,344 ± 23,371 IU/L at 6 h, and 23,371 ± 11,660 IU/L at 24 h (Fig 1E). CK is a marker of plasma membrane damage and cell death of skeletal muscle fibers; hence it appears that myotoxic activity of the venom is highest at one hour, decreasing afterwards. These results are in agreement with the muscle tissue damage observed in the histological analysis, which occurs early on in the course of envenoming.

### Proteomic analysis of wound exudates

Wound exudate samples collected from mice injected with *B*. *asper* venom were decomplexed by SDS-PAGE for subsequent proteomic analysis. From the mass spectrometric analysis of the gel bands, a total of 537, 578, and 486 proteins were identified in exudates at 1 h, 6 h, and 24 h, respectively, with protein identification probability above 95% and minimum of two peptides (S1 Table). The most abundant proteins identified based on their Quantitative Value (see http://www.proteomesoftware.com/ for full description of term) were classified within the following groups: intracellular proteins (Table 1 and S2 Table), ECM proteins (Table 2), membrane proteins (S3 Table), coagulation factors (S4 Table), and proteinase inhibitors (S5 Table). Within each group, the proteins were organized by those that changed at least three fold as compared to another time and proteins which did not show significant change between the three time intervals, i.e. those whose amounts did not differ more than threefold between times.

**Table 1:** More abundant intracellular proteins identified in wound exudates collected from mice at 1, 6 and 24 h after injection of *B*. *asper* venom, which changed at least three fold at one time as compared to another time.

Protein	Accession Number	Molecular mass	Quantitative Value		
			1h	6h	24h
Creatine kinase M-type	P07310	43 kDa	651	404	96
Fructose-bisphosphate aldolase	A6ZI44	45 kDa	357	216	109
Phosphorylase	E9PUM3	88 kDa	300	294	47
Carboxylesterase 1C	P23953	61 kDa	200	168	0
Alpha-actinin-2	Q9JI91	104 kDa	190	298	59
Alpha-actinin-3	O88990	103 kDa	161	206	55
Actin, alpha skeletal muscle	P68134	42 kDa	120	258	220
L-lactate dehydrogenase	G5E8N5	40 kDa	105	87	34
Triosephosphate isomerase	P17751	32 kDa	98	96	27
Bisphosphoglycerate mutase	O70250	29 kDa	93	15	14
Cofilin-1	P18760	19 kDa	83	10	92
Peroxiredoxin-5, mitochondrial	P99029 [2]	22 kDa	83	16	17
Glutathione peroxidase 1	P11352	22kDa	83	13	14
Isoform 2 of Myc box-dependent-interacting protein 1	O08539-2 [2]	48 kDa	83	22	0
Sarcoplasmic/endoplasmic reticulum calcium ATPase 1	Q8R429 [2]	109 kDa	80	77	0
UTP--glucose-1-phosphate uridylyltransferase	Q91ZJ5	57 kDa	74	78	11
Myosin-binding protein H	P70402	53 kDa	74	67	0
Ubiquitin-40S ribosomal protein S27a	P62983 [2]	18 kDa	74	15	26
Flavin reductase (NADPH)	Q923D2	22 kDa	74	15	22
L-lactate dehydrogenase B chain	P16125	37 kDa	65	18	80
Malate dehydrogenase, mitochondrial	P08249	36 kDa	65	63	14
Myosin-9	Q8VDD5	226 kDa	65	10	31
Phosphoglycerate kinase 1	P09411	45 kDa	58	47	15
Heat shock cognate 71 kDa protein	P63017 [4]	71 kDa	51	73	17
Aconitate hydratase, mitochondrial	Q99KI0	85 kDa	48	99	10
Thioredoxin	P10639	12 kDa	46	16	92
Isoform 3 of Elongation factor 1-delta	P57776-3	73 kDa	46	78	23
Isoform Cytoplasmic of Fumarate hydratase, mitochondrial	P97807-2	50 kDa	46	15	69
Myosin-4	Q5SX39 [9]	223 kDa	43	620	529
Isocitrate dehydrogenase [NAD] subunit alpha, mitochondrial	Q9D6R2	40 kDa	37	67	0
Glucose-6-phosphate isomerase	P06745	63 kDa	31	52	17
Ubiquitin-like protein ISG15	Q64339	18 kDa	28	90	34
3-ketoacyl-CoA thiolase, mitochondrial	Q8BWT1	42 kDa	28	90	11
Protein disulfide-isomerase	P09103	57 kDa	28	17	69
Superoxide dismutase [Cu-Zn]	P08228	16 kDa	28	10	57
L-lactate dehydrogenase C chain	P00342	36 kDa	26	20	80
Peptidyl-prolyl cis-trans isomerase A	P17742	18 kDa	22	67	24
Adenylate kinase isoenzyme 1	Q9R0Y5 [2]	22 kDa	20	78	23
Myosin regulatory light chain 12B	Q3THE2	20 kDa	19	34	57
Isocitrate dehydrogenase [NADP], mitochondrial	P54071	51 kDa	19	78	0
Cofilin-2	P45591	19 kDa	17	78	46
Clathrin heavy chain 1	Q68FD5	192 kDa	17	18	92
Glutathione S-transferase P 1	P19157	24 kDa	16	15	80
Elongation factor 1-gamma	Q9D8N0	50 kDa	16	22	69
Carboxypeptidase N catalytic chain	Q9JJN5	52 kDa	12	90	92
GTP-binding nuclear protein Ran, testis-specific isoform	Q61820 [2]	24 kDa	11	34	80
Myosin light chain 1/3, skeletal muscle isoform	P05977 [2]	21 kDa	10	26	59
Succinate dehydrogenase [ubiquinone] iron-sulfur subunit, mitochondrial	Q9CQA3	32 kDa	1	78	92
ATP synthase subunit beta, mitochondrial	P56480	56 kDa	1	34	80
Tropomyosin beta chain	P58774	33 kDa	1	78	33

**Table 2:** Extracellular matrix proteins identified in wound exudates collected from mice at 1, 6 and 24 h after injection of *B*. *asper* venom.

Protein	Accession Number	Molecular mass	Quantitative value		
			1 h	6 h	24 h
**Proteins which changed at least three fold at one time as compared to another time**					
Basement membrane-specific heparan sulfate proteoglycan core protein	B1B0C7 [2]	469 kDa	83	56	0
Collagen alpha-1(XVIII) chain	E9QPX1 [2]	182 kDa	74	22	0
Thrombospondin-1	P35441	130 kDa	65	16	11
Protein Col6a3	E9PWQ3	354 kDa	56	45	0
Collagen alpha-1(XV) chain	A2AJY2 [2]	138 kDa	56	45	0
Collagen alpha-2(IV) chain	P08122	167 kDa	37	11	0
Collagen alpha-1(IV) chain	P02463	161 kDa	19	11	0
Nidogen-2	O88322	154 kDa	19	11	0
Nidogen-1	P10493	137 kDa	19	56	0
Collagen alpha-1(III) chain	P08121	139 kDa	12	45	23
Collagen alpha-2(I) chain	Q01149	130 kDa	16	22	57
Collagen alpha-1(I) chain	P11087	138 kDa	15	13	57
Laminin subunit gamma-1	P02468 [2]	177 kDa	1	56	46
Fibrillin-2	Q61555	314 kDa	1	22	0
Chondroitin sulfate proteoglycan 4	Q8VHY0	252 kDa	0	22	11
Fibrillin-1	A2AQ53 [2]	312 kDa	0	22	0
Collagen alpha-1(XII) chain	E9PX70 [3]	334 kDa	0	22	0
**Proteins which did not change more than three-fold at any time as compared to another time**					
Fibronectin	P11276	273 kDa	275	194	291
Thrombospondin-4	Q9Z1T2	106 kDa	65	34	46
Vitronectin	P29788	55 kDa	19	13	10
Dermatopontin	Q9QZZ6	24 kDa	19	11	23
Proteoglycan 4	E9QQ17 [4]	111 kDa	19	11	13
Collagen alpha-1(XIV) chain	B7ZNH7	193 kDa	12	16	29
Lumican	P51885	38 kDa	11	11	17

A total of 222 intracellular proteins (Table 1 and S2 Table) and 13 membrane proteins (S3 Table) were detected in exudates, thus demonstrating direct or indirect cellular damage induced by the venom. The most abundant intracellular proteins detected in exudates were hemoglobin subunit beta-2 and creatine kinase M-type, in agreement with the hemorrhagic and myotoxic activity of *B*. *asper* venom, respectively. Moreover, the creatine kinase M-type identified in the exudates was detected at the highest level at 1 h, and decreased over time until reaching a six fold reduction at 24 h. These results are in agreement with the CK activity of exudates and the muscle tissue damage observed in the histological analysis. In contrast, there was a trend for cytoskeletal proteins, such as actin, myosin, and tropomyosin, to increase in the exudates over time, while most of cytosolic and mitochondrial proteins appeared at the first hour of venom injection, and decreased afterwards.

Of serum proteins, a total of 10 coagulation factors (S4 Table) and 14 proteinase inhibitors (S5 Table) were detected in exudates at various times. Fibrinogen beta and gamma chains appeared in the exudates at the first hour following venom injection and their abundance increased over time. Other coagulation factors detected whose amounts changed at least three fold as compared to values at other time were coagulation factor X, XII, and XIII. The inter alpha-trypsin inhibitor was the only proteinase inhibitor that increased at least threefold at 24 h as compared to 1 h and 6 h.

**Fig 2:**
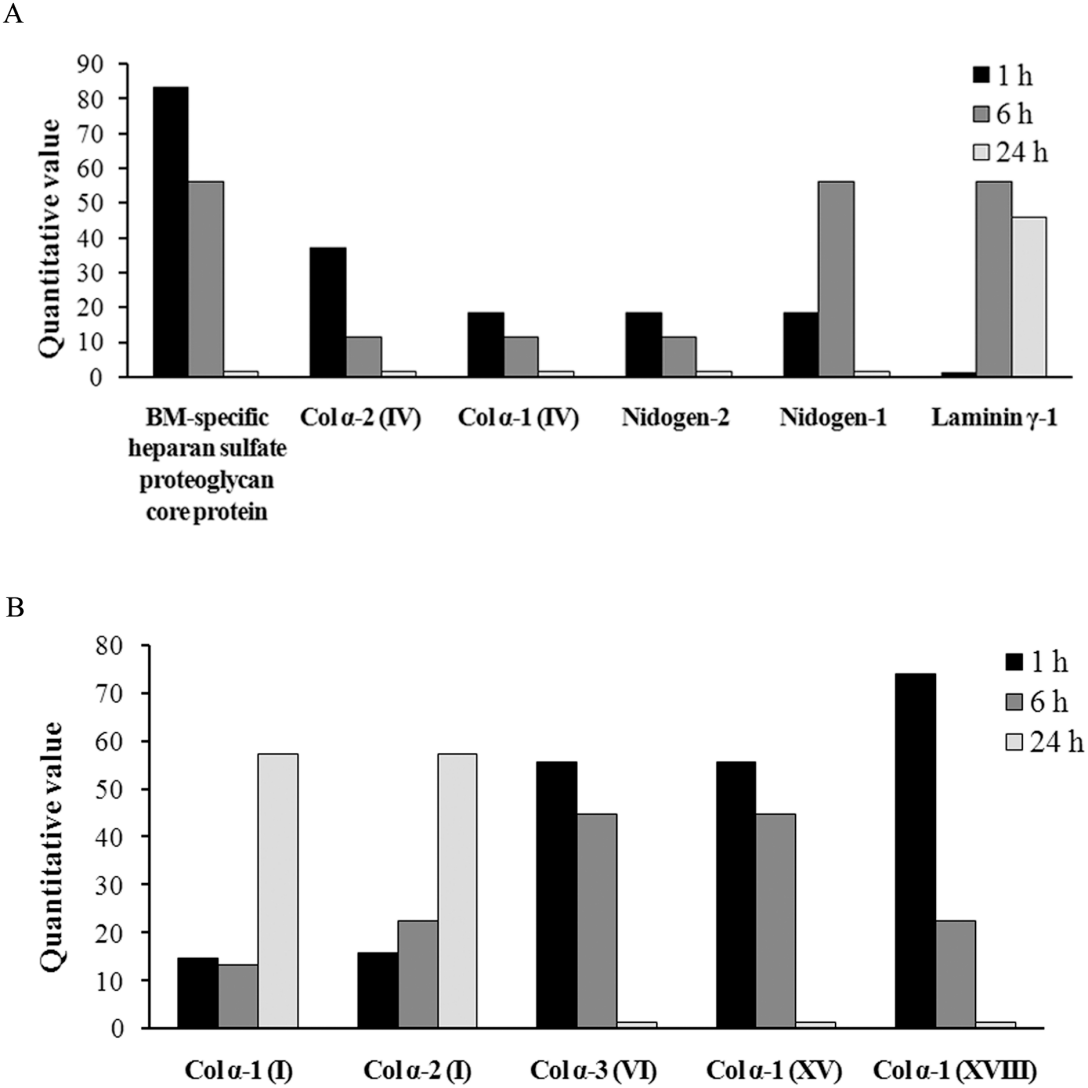
Extracellular matrix proteins identified in wound exudates collected from mice after injection of *B*. *asper* venom. Groups of five mice were injected in the gastrocnemius with 50 μg of *B*. *asper* venom. After 1, 6 and 24 h of injection, mice were sacrificed and samples of exudate were collected, pooled and lyophilized. Proteomic analysis of exudates was performed as described in Methods. Proteins from the BM are included in (A), whereas other ECM proteins are depicted in (B). Only proteins whose amount varied at least three fold between time intervals were included in this figure. Notice that the level of BM proteins and non-fibrillar collagens are higher at 1 h, whereas type I collagen levels are higher at 24 h.

A total of 24 ECM proteins were identified in exudates, of which 21, 24, and 13 proteins were detected at 1 h, 6 h, and 24 h, respectively (Table 2). Most of these proteins showed a differential abundance greater than three-fold between samples collected at different times. The most abundant basement membrane (BM) protein detected in the wound exudates was BM-specific heparan sulfate proteoglycan core protein (perlecan), followed by alpha 1 and 2 chains of type IV collagen. Most of the BM components, such as heparan sulfate proteoglycan, type IV collagen and nidogen-2, appeared in the exudates at 1 h, and the amount decreased over time, largely becoming undetectable at 24 h. Conversely, the amount of laminin γ-1 detected in the exudates increased at 6 h and 24 h, and the amount of nidogen-1 increased at 6 h as compared to 1 h and 24 h (Fig 2A). Other collagens, such as types VI, XV, and XVIII collagens, were present in the exudates at 1 h and 6 h, but were not detected at 24 h. Interestingly, type I collagen was also detected in the exudates at the first hour and its abundance increased at 24 h (Fig 2B). Other ECM proteins detected in the exudates whose abundance were greater at 6 h as compared to 1 h and 24 h were type III collagen, fibrillin 1 and 2, chondroitin sulfate proteoglycan 4, and type XII collagen. On the other hand, thrombospondin 1 appeared in the exudates at 1 h and decreased over time. Other ECM proteins detected in the exudates whose amounts did not vary more than threefold between times were fibronectin, thrombospondin-4, vitronectin, dermatopontin, proteoglycan 4, type XIV collagen, and lumican (Table 2).

### Immunochemical detection of ECM proteins in wound exudates

#### Type IV collagen

**Fig 3:**
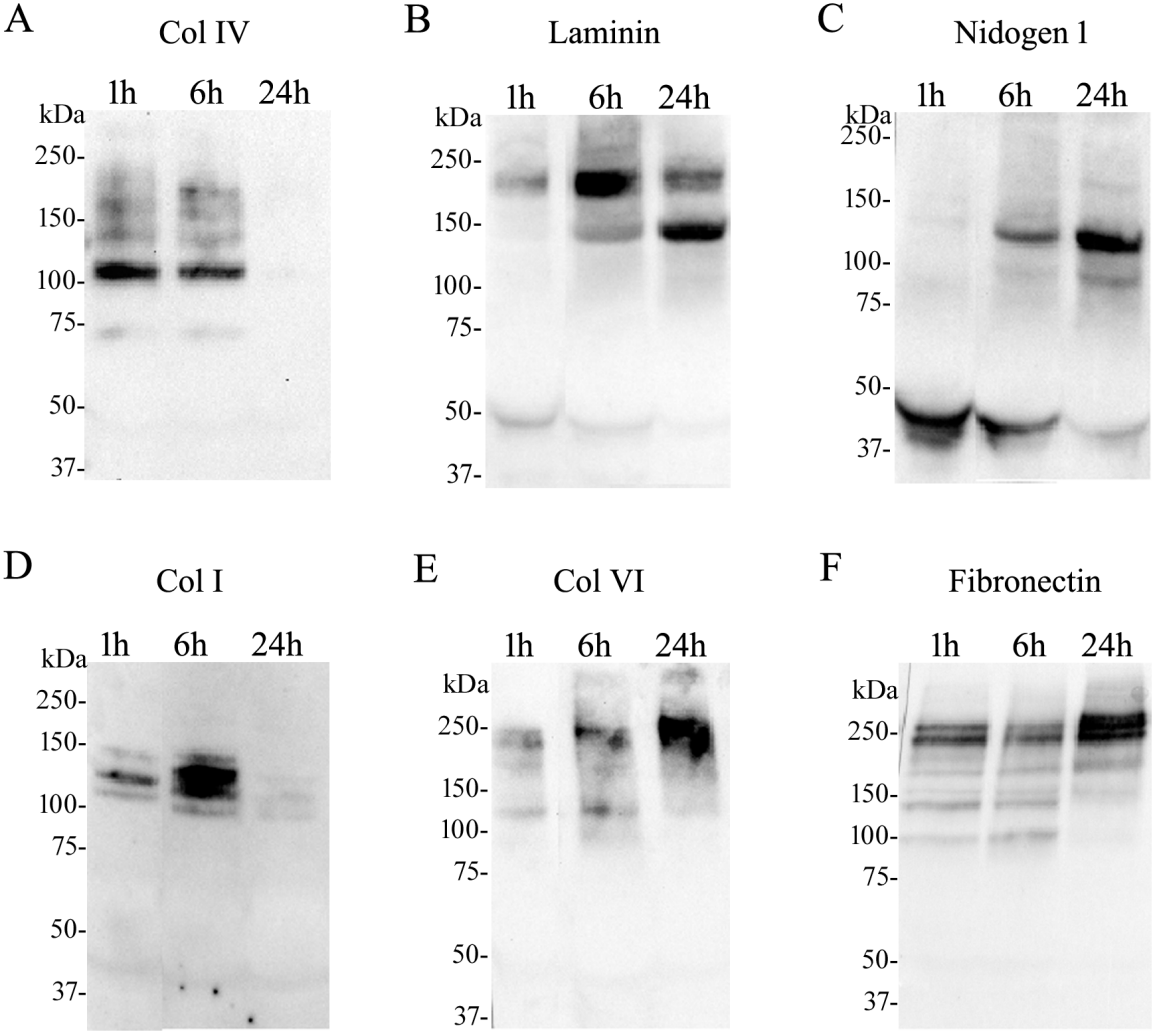
Western blot analysis of extracellular matrix components in wound exudates collected from mice after injection of *B*. *asper* venom. Groups of five mice were injected in the gastrocnemius with 50 μg of *B*. *asper* venom. After 1, 6 and 24 h of injection mice were sacrificed and samples of exudates were collected, pooled and lyophilized. Afterwards, 100 μg of protein of each sample were separated under reducing conditions on 4–15% Tris–HCl SDS-PAGE, and transferred to nitrocellulose membranes. Immunodetection was performed with (A) anti-type IV collagen (Col IV), (B) anti-laminin, (C) anti-nidogen 1, (D) anti-type I collagen (Col I), (E) anti-type VI collagen (Col VI), and (F) anti-fibronectin. The reaction was detected using an anti-rabbit peroxidase antibody and a chemiluminescent substrate. Images were obtained with the ChemiDoc XRS+ System (BioRad).

Immunodetection of type IV collagen in wound exudates showed one prominent band of 107 kDa, with additional faint bands of 217 kDa, 172 kDa, 135 kDa and 70 kDa at 1 h and 6 h (Fig 3A). A reduction of intensity of the 107 kDa band was observed at 6 h as compared to 1 h. The molecular mass of the prominent band does not agree with the molecular mass of the alpha chains of type IV collagen (145–160 kDa). Thus, these bands very likely constitute degradation products. Type IV collagen and its degradation products were not detected in exudate samples collected at 24 h. Therefore, according to Western blot results, there is a rapid degradation of type IV collagen within the first 6 hours.

#### Laminin

Immunodetection of laminin showed the presence of this protein in the wound exudates at 1, 6, and 24 h with two prominent bands of 220 kDa and 140 kDa (Fig 3B). These bands agree with the molecular mass of some isoforms of alpha, beta, and gamma chains (130–200 kDa) of this protein. A reduction of intensity of the 220 kDa band was observed at 1 h as compared to 6 h and 24 h. The band of 140 kDa was not detected at 1 h. A reduction of intensity of the 220 kDa band and an increase of the intensity of the 140 kDa band were observed at 24 h as compared to 6 h.

#### Nidogen 1

Western blot analysis of wound exudates for nidogen 1 showed two prominent bands of 120 and 40 kDa (Fig 3C), which are likely to be degradation fragments. A reduction of the 40 kDa band with an increase of the 120 kDa band was observed over time.

#### Type I collagen

Immunodetection of type I collagen showed three bands corresponding to proteins of 135 kDa, 120 kDa and 107 kDa at 1 h and 6 h (Fig 3D). An increase in the intensity of these bands, in particular the 120 kDa, was observed at 6 h as compared to 1 h. These bands agree with the molecular mass of some isoforms of alpha 1 (120 kDa) and alpha 2 (115 kDa) chains. Collagen I and degradation products were not detected at 24 h in the exudates. Overall there is a trend of increasing abundance of type I collagen in the exudates by Western blot analyses at 6 h as compared to 1 h.

#### Type VI collagen

Immunoblotting of wound exudates for type VI collagen showed one prominent band of 230 kDa, and a faint band of 118 kDa at 1 h and 6 h (Fig 3E). The presence of a band of 118 kDa in the first 6 h indicates degradation of type VI collagen early in the course of envenoming. A reduction of the 118 kDa band, with an increase of the 230 kDa band, was observed over time. Therefore, according to Western blot results, type VI collagen is degraded in the first hours, and then type VI collagen increases and appears in the exudates collected at 24 h.

#### Fibronectin

Immunodetection of fibronectin showed two bands of 265 kDa and 236 kDa, with additional faint bands of 175 kDa, 140 kDa, and 100 kDa at 1 h, 6 h and 24 h (Fig 3F). An increase of intensity of the most abundant bands and a reduction of the faint bands were observed at 24 h as compared to 1 h and 6 h. The molecular mass of the 265 kDa band agrees with the molecular mass expected (262 kDa). Therefore, according to Western blot results, fibronectin is degraded in the first hours, but it remains present at 24 h in the exudates.

### Proteolytic activity of wound exudates

**Fig 4:**
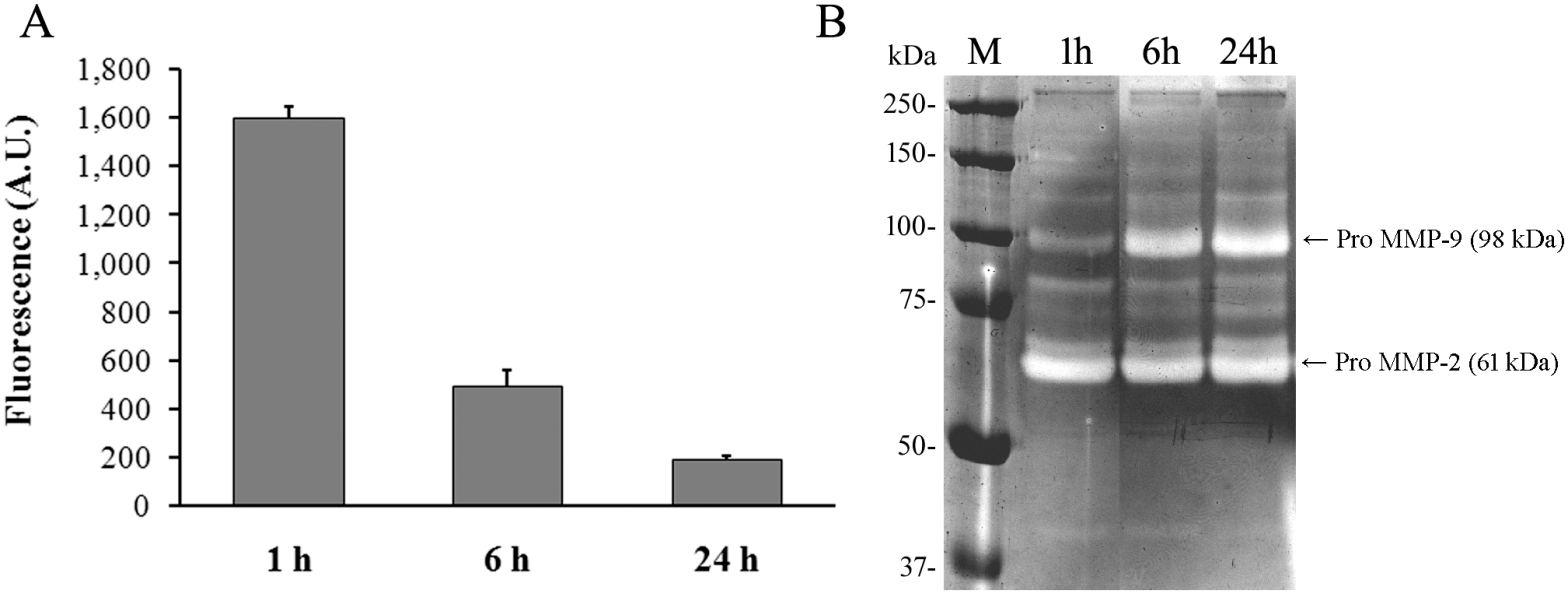
Proteolytic activity of wound exudates collected from mice after injection of *B*. *asper* venom. Groups of five mice were injected in the gastrocnemius with 100 μg of *B*. *asper* venom. After 1, 6 and 24 h, mice were sacrificed and samples of exudates were collected. (**A**) Proteolytic activity of exudate samples was measured after 24 h of incubation with gelatin fluorescein conjugate using a commercial kit (EnzCheck protocol Gelatinase/Collagenase Assay Kit, Molecular Probes, Life Technologies) as described in Methods. Results are expressed as mean ± S.D (n = 3) of Fluorescence Arbitrary Units (A.U.). (**B**) Exudate samples were separated in a 7.5% SDS-polyacrylamide gel containing 0.50 mg/mL of Type A gelatin. The gel was incubated at 37°C and then stained with Coomassie blue R-250. MMP: matrix metalloproteinase (see text for explanation); M: lane corresponding to molecular mass markers.

In order to determinate whether SVMP or endogenous proteases are active in the wound exudates, proteolytic activity assays of exudate samples were performed. Exudate samples collected from mice injected with *B*. *asper* venom at 1 h showed the highest proteolytic activity on gelatin fluorescein conjugate compared with samples collected at 6 h and 24 h. (Fig 4A). When exudates collected at 1 h were incubated with polyclonal antibody against the SVMP BaP1, the proteolytic activity of the exudates was almost completely inhibited since only 8% of the activity remained.

Using zymography several gelatinolytic bands were detected corresponding to proteins of 50–150 kDa in the exudate samples collected at different times (Fig 4B). An increase of two main bands of about 100 kDa and 60 kDa was observed in exudates collected at 6 h and 24 h. These molecular masses are consistent with the latent forms of matrix metalloproteinases (MMP) 9 and 2, respectively [22]. Therefore, the zymography showed an increase of proteolytic activity of endogenous MMPs in exudate over time. Furthermore, bands corresponding to the molecular mass of the SVMP BaP1 were not detected in the zymographic analysis of exudate samples.

## Discussion

Envenoming by venomous snakes gives rise to a complex pathophysiology by virtue of the complexity of the venoms and the fact that the toxins in the venom produce manifold effects in the tissues. Proteomic analysis of wound exudates collected in the vicinity of affected tissue constitutes a powerful approach to study the pathogenesis of tissue damage induced by snake venoms from a more comprehensive perspective [13–17], thus complementing histological, ultrastructural and biochemical analyses. This methodological tool has been used to investigate the early alterations provoked by *B*. *asper* venom [15] and some of its toxins, especially myotoxic PLA_2_s and hemorrhagic SVMPs [13,14,16], as well as the inhibitory effects of antivenoms and low molecular mass inhibitors [15]. However, analyses in these previous studies were performed at a single time interval after injection, thus precluding the understanding of these events from a time-course perspective. In this study, we investigated the dynamics of local effects induced by *B*. *asper* venom in the gastrocnemius muscle of mice at various time intervals.

Our histological and biochemical observations agree with previous studies showing a rapid development of myonecrosis and hemorrhage, followed by an inflammatory process characterized by the infiltration of neutrophils and macrophages at later time intervals [4,5,7,9,10]. Previous works on venom-induced myonecrosis have quantified CK activity in plasma [6], where the highest levels were observed at 3 h post envenoming. In contrast, in exudates, highest CK levels are higher than in plasma, and peak activity occurs at 1 h instead of 3 h. This difference may be attributed to the kinetics of absorption of this enzyme into the circulation after its release from damaged muscle fibers, since exudate was collected close to the venom-injected muscle. In agreement with previous pathological and proteomic studies, intracellular proteins were abundant in exudates, as a consequence of the cytotoxic effect of venom, especially on skeletal muscle fibers [7,11,14,15]. The time-course analysis of the intracellular proteins in exudate underscores that most of the cytosolic and mitochondrial proteins appear early on due to the rapid action of myotoxic PLA_2_s and PLA_2_ homologues in muscle tissue [7,11,14,15,23], followed by a decrease of these proteins. Most of these proteins are derived from the cytosol of skeletal muscle fibers since myotoxic PLA_2_s induce a rapid disruption of the integrity of muscle cell plasma membrane [4,7,24]. The high CK activity of exudates at 1 h and our histological observations corroborate the early onset of myonecrosis in the course of envenoming and agree with proteomic analysis.

In contrast to cytosolic proteins, most of the cytoskeletal proteins, such as actin, myosin, and tropomyosin, are more abundant in exudates collected at later time periods. This late increment suggests that the presence of cytoskeletal protein fragments in the exudate depends on the action of proteinases that release these structural components from damaged cells. A prominent calcium influx in muscle cells occurs after venom-induced plasma membrane damage [25,26]. An increased calcium concentration in the cytosol results in the activation of calpains, which might hydrolyze cytoskeletal components [27]. Subsequently, proteinases derived from inflammatory cells arriving at the necrotic tissue may also contribute to proteolysis of muscle cytoskeletal proteins [9,10]. Thus, the proteomic analyses reveals two ‘waves’ of release of intracellular proteins to exudates: an early release of cytosolic and mitochondrial proteins, which depends on the rapid myotoxin-induced membrane damage, and a more delayed release of cytoskeletal protein fragments, which is due to proteolytic degradation.

The presence of cell membrane-associated proteins may be evidence of direct or indirect cellular damage induced by the venom. Moreover, proteolysis of these components, either by venom or endogenous proteinases, may cause their ‘shedding’ and diffusion to the exudate compartment. It is tempting to speculate that, in addition to being a passive reflection of venom-induced plasma membrane damage, the release of these protein fragments may also play a functional role in cellular signaling associated with inflammatory and reparative events. However, the pathological relevance of the hydrolysis of these proteins in the overall mechanism of local tissue damage induced by snake venoms has not been established and needs further study.

The presence of ECM proteins in wound exudates reflects the cleavage by either venom-derived proteinases or endogenous proteinases, such as MMPs, generated in the course of the inflammatory response. The degradation of ECM is a relevant component of viperid venom-induced tissue damage, and proteomic analysis has been particularly useful in revealing a complex pattern of hydrolysis [14–16]. Previous studies detected *B*. *asper* venom components in muscle homogenates of mice during the first week after experimental envenoming [28]; however, the activity of these toxins has not been previously addressed. When assessing the proteinase activity of exudates, highest activity was detected in samples collected after 1 h of envenoming; here we demonstrate that this is mainly due to the action of SVMPs, since antibodies against BaP1, the most abundant proteinase in *B*. *asper* venom [29], almost fully inhibited exudate-induced proteolysis. However, this enzymatic activity decreased over time, probably as a consequence of diffusion of venom components from the injected muscle or of inhibition by plasma or tissue-derived proteinase inhibitors. It is likely that activity at later time intervals, i.e. 24 h, is mostly due to endogenous MMPs generated in the course of the inflammatory response, such as MMP-9 and MMP-2, which was confirmed by the detection with zymography of the wound exudates, although it remains possible that some venom proteinases persisting in the tissue may also contribute to this observation. These results agree with previous studies which demonstrated an increase in the expression of MMP-9 and MMP-2 in muscle tissue injected with *B*. *asper* venom [28] or with purified SVMP and PLA_2_ toxins [30]. Taken together these findings suggest that the hydrolysis of ECM is mainly due to SVMPs in the early stages of envenoming, while endogenous MMPs participate later in the course of envenoming.

A large body of experimental evidence indicates that BM and related ECM components that provide stability to microvessel structure are the key targets of hemorrhagic SVMP [5,13,14,16,31–34]. Moreover, SVMP-induced hemorrhage occurs very fast after injection [12,35–37]. Therefore, the presence of ECM components in wound exudates during first hour as compared to later time periods may offer important insights for understanding the mechanism of action of hemorrhagic SVMPs. Regarding BM components, the presence of degradation products of perlecan, type IV collagen, nidogen, and laminin in wound exudates underscores a rapid and drastic damage of BM structure. In particular, perlecan and type IV collagen are abundant in exudates after the first hour, when hemorrhagic events have occurred, and then their amounts decrease over time, as they were not detected by proteomic analysis at 24 h. Western blot analysis of exudate confirmed the presence of fragments of type IV collagen 1 h after venom injection.

Perlecan is the most abundant BM protein detected in the wound exudates during the first hour. In previous proteomic studies, we have found that the relative amount of perlecan in wound exudates induced by a hemorrhagic SVMP was greater as compared to a non-hemorrhagic one [13], but similar when compared to other hemorrhagic SVMPs [16], and its presence was abolished when *B*. *asper* venom was previously incubated with batimastat [15], a metalloproteinase inhibitor. Such findings, together with our data, suggest that degradation of perlecan in early stages of envenoming may play an important role in the hemorrhagic mechanism of SVMPs. This proposal agrees with the known structural role of perlecan in BM [38–42]. In addition, mutations in the perlecan gene in mice have been associated with loss of BM integrity in different tissues [43–45], including microvasculature of brain and skin, which cause severe bleedings due to dilatation and rupture of microvessels [45].

On the other hand, previous proteomic studies using similar models have not detected type IV collagen in wound exudates induced by *B*. *asper* venom or its toxins [13–16]. However, several studies using *in vitro* and *in vivo* models have identified type IV collagen as one of the most likely key components degraded by hemorrhagic SVMPs and associated with the initial microvessel destabilization and hemorrhage [5,13,16,34,46,47]. Our present proteomic results did identify fragments of type IV collagen in exudates at 1 h, thus agreeing with previous immunohistochemical and immunochemical evidence [13,16]. In addition, according to Western blot analysis, degradation products of type IV collagen appear in exudates in samples collected at 1 h. Such early appearance of type IV collagen and perlecan strongly suggest that their degradation is due to the direct proteolytic activity of SVMPs. The hypothesis that type IV collagen is a key target in the hemorrhagic mechanism of SVMPs is compatible with the structural role of this collagen in the mechanical stability of BM, as it is stabilized by covalent cross-links [41,42,48–51]. In addition, mutations on type IV collagen genes have been associated with pathological alterations in microvessels and with hemorrhage in brain, kidney and lungs in mice and humans [52–58]. Thus, the rapid hydrolysis of perlecan and type IV collagen after injection of *B*. *asper* venom supports the view that BM destabilization leading to hemorrhage is likely to depend on the degradation of these mechanically-relevant components.

Nidogen 2 appeared in early time periods in wound exudates, in agreement with previous proteomic studies [13,15], and then it decreased over time in our proteomics analysis. Since nidogen 2 is more abundant in the BM of blood vessels [59], and its time-course dynamics of appearance in exudates is similar to that observed for type IV collagen and perlecan, the release of nidogen 2 might be associated with vascular BM damage. In contrast, taken together the proteomic and Western blot analyses showed that nidogen 1 increased over time in wound exudates. In addition, nidogen 1 and 2 have been detected in plasma of healthy mice [60], which could explain the presence of nidogen 1 in the wound exudates according to Western blot results. On the other hand, laminin γ1, which is widely distributed [61,62], also increased over time in wound exudates. The time-course variation of the molecular masses of immunoreactive bands in the cases of nidogen 1 and laminin underscores the dynamics of degradation of these components over time. Furthermore, Escalante et al. [13] demonstrated similar patterns of degradation for nidogen and laminin in muscle tissue induced by hemorrhagic and non-hemorrhagic SVMPs.

Our observations allowed the analysis of the time-course dynamics of the hydrolysis of non-fibrillar collagens associated with the BM, such as types VI, XV, and XVIII collagens. As in the case of type IV collagen and perlecan, hydrolysis of these components was highest at 1 h, hence indicating a rapid degradation, probably by venom proteinases. These collagens connect the BM with fibrillar collagens of the matrix [39,63], and are known to play a relevant role in the mechanical stability and integration of BM with connective tissue [39,63]. Hence, the hydrolysis of these components by SVMP might be also critical for capillary wall destabilization, as have been previously proposed [13,64]. Alternatively, the increase of these collagens in exudates might be consequence of BM damage after the hydrolysis of other components, such as type IV collagen and perlecan. Type VI collagen is more abundant in the BM of muscle cells [65–67]; thus the increment of non-degraded type VI collagen chains in exudates could reflect synthesis *de novo* during reparative and regenerative events in muscle tissue. The role of the degradation of these collagens in the initial destabilization of BM induced by hemorrhagic SVMP is an issue that should be further investigated.

Other ECM components of interest detected in the proteomic analysis are collagen I and fibronectin. Collagen I is a fibril-forming collagen distributed in non-cartilaginous connective tissues such as skin and connective tissue of muscle [63]. According to proteomic results, the relative abundance of collagen I in exudates is higher at 24 h as compared to 1 and 6 h. This late hydrolysis of collagen I could be result of the action of endogenous MMPs synthesized during the course of inflammation in the damage tissue. Fibronectin was detected in the exudates both in proteomic and immunochemical analyses. This protein can be found in two forms: plasma fibronectin, which is a soluble molecule synthesized by hepatocytes, and cellular fibronectin, which is produced in the tissues and is incorporated in the ECM [62]. Thus, the presence of fibronectin in exudates could be either a consequence of plasma exudation or hydrolysis from the ECM. According to proteomic analysis, the amount of fibronectin in exudates does not change over time; however, on the basis of Western blot analysis, it appears to be more degraded at early time periods most likely due to the action of SVMPs.

Taken together, our observations highlight a dual pattern of ECM protein degradation and appearance in exudates. Types IV and VI collagens, perlecan, nidogen and fibronectin show a higher degradation early on in the course of envenoming, correlating with the rapid action of SVMPs upon venom injection, as demonstrated by the proteinase activity of exudates. The rapid action of SVMPs on various key components of the BM is likely to be causally related to microvessel damage and hemorrhage. In the case of the fibrillar collagen I, it seems to be degraded predominantly by endogenous MMPs at later time periods, during the inflammatory reaction that ensues in the tissue as a consequence of venom-induced damage, as evidenced by zymography.

The presence of abundant plasma proteins in the exudate, as revealed by proteomic analysis, is a consequence of plasma exudation as a result of edema and increment in vascular permeability induced by the venom [68,69]. Some of the plasma proteins detected are acute-phase proteins, proteinase inhibitors and coagulation factors. Of interest is the increase of fibrinogen and the inter α-trypsin inhibitor heavy chains over time.

The presence of fibrinogen in exudates might be secondary to the inflammatory exudation induced by the venom since this protein is typically found in plasma at high concentrations [70]. Previous proteomic studies have found fibrinogen in the wound exudates induced by *B*. *asper* venom and its toxins, especially SVMP BaP1, early in the course of envenoming [14,15]. Our data show an increase of fibrinogen in wound exudates over time. This increment might be consequence of fibrin clot formation in capillary walls, due to vascular damage induced by SVMPs [12,14,35], and also to fibrin formation in the extravascular interstitial space, followed by fibrinolysis by endogenous proteinases [70], thus explaining their higher amounts in exudates collected at later time intervals.

The inter-α-trypsin inhibitor heavy chains are mainly secreted into the blood by the liver as serum protease inhibitor whose concentration increases in inflammatory conditions [71]. The effect of these proteins in tissues has been associated with both inflammatory and anti-inflammatory activities [71–73]. Moreover, these proteins can be covalently linked to hyaluronan, exerting functions on cell migration and ECM remodeling under physiological and pathological conditions [74,75]. Thus, the increase of inter-α-trypsin inhibitor heavy chains detected in exudates might be due to an acute-phase inflammatory response and to the tissue inflammation as a consequence of venom-induced damage.

**Fig 5:**
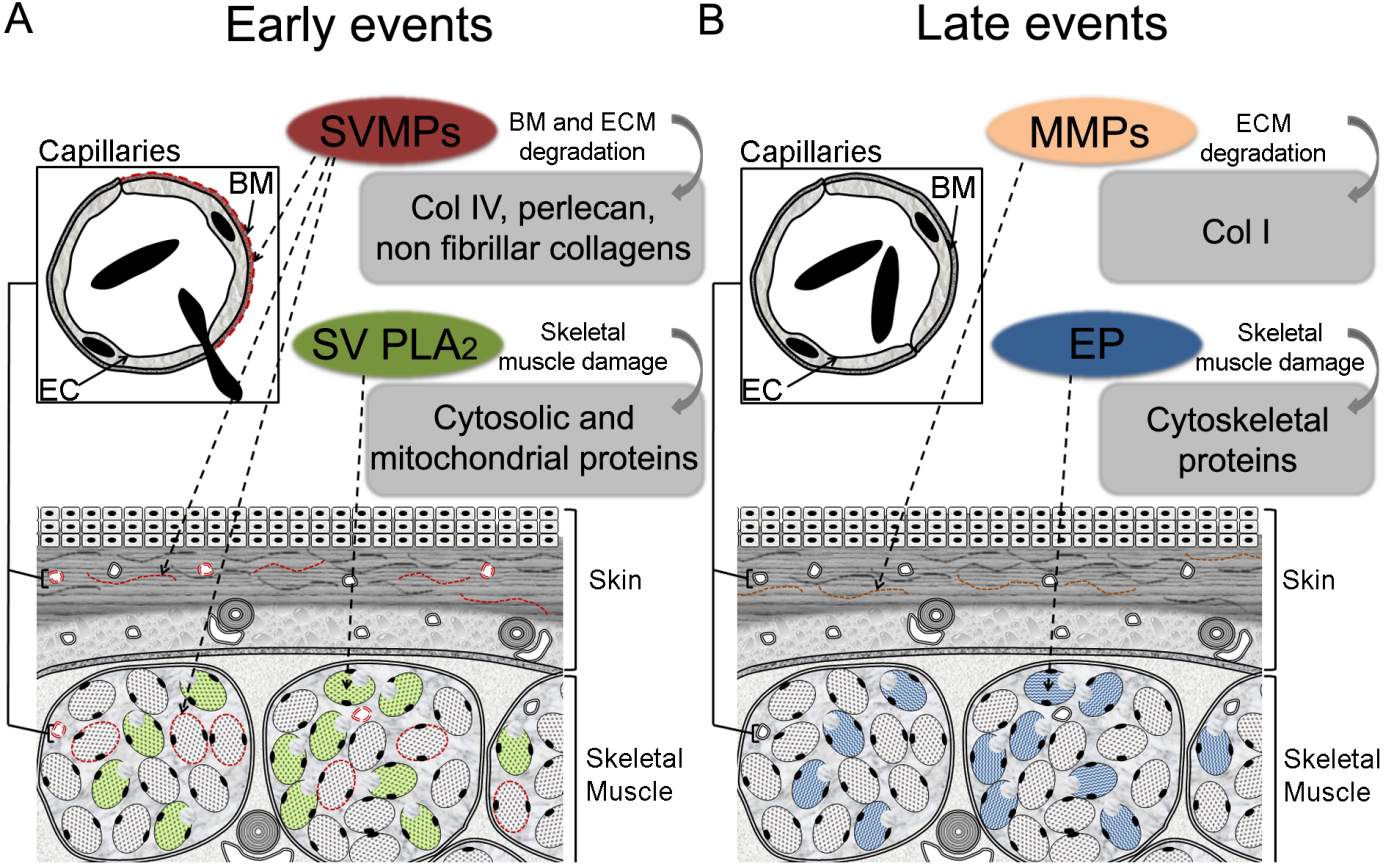
Early and late pathological events induced by the venom of *Bothrops asper* in muscle tissue. (**A**) The venom of *B*. *asper* induces a rapid cytotoxic (especially myotoxic) and hemorrhagic effects evidenced by the early release of cytosolic and mitochondrial proteins, and the degradation of BM and related ECM, respectively. (**B**) Then, MMPs and other endogenous proteinases are associated with tissue remodeling and degradation of cytoskeletal proteins, especially in skeletal muscle, as part of the inflammatory reaction later on in the course of envenoming. BM: basement membrane; EC: endothelial cells; ECM: extracellular matrix; SVMPs: snake venom metalloproteinases; SV PLA_2_s: snake venom phospholipases A_2_; EP: endogenous proteases.

In conclusion, the proteomic analysis of wound exudates performed in this study provides a more complete understanding of the time-course dynamics of muscle tissue damage induced by *B*. *asper* venom. These observations, together with Western blot and histology data, provide a more integrated view of venom-induced local tissue damage (Fig 5). The early presence of cytosolic and mitochondrial proteins in exudates, as compared to the later increase of cytoskeletal proteins, confirms the rapid cytotoxic effect of venom, followed by the action of endogenous proteinases in the cytoskeleton of damaged muscle fibers. On the other hand, the early presence of BM and other BM-associated ECM components in exudates, together with venom-derived proteolytic activity of exudates, strongly suggest the hydrolysis of these components by SVMPs in the early stages of envenoming. In contrast, the increment of some ECM proteins in the exudates at later time intervals is likely to be due to the action of endogenous MMPs or to their synthesis *de novo* during tissue remodeling associated with inflammation and reparative processes. Finally, the time-course of appearance in wound exudates of type IV collagen and perlecan supports the role of the hydrolysis of these BM components in the mechanism of microvascular damage induced by hemorrhagic SVMP.

## Supporting Information

S1 Table List of all proteins identified in wound exudates collected from mice at 1, 6 and 24 h after injection of *B*. *asper* venom. (PDF)

S2 Table Intracellular proteins identified in wound exudates collected from mice at 1, 6 and 24 h after injection of *B*. *asper* venom. (PDF)

S3 Table Membrane proteins identified in wound exudates collected from mice at 1, 6 and 24 h after injection of *B*. *asper* venom. (PDF)

S4 Table Coagulation factors identified in wound exudates collected from mice at 1, 6 and 24 h after injection of *B*. *asper* venom. (PDF)

S5 Table Serum proteinase inhibitors identified in wound exudates collected from mice at 1, 6 and 24 h after injection of *B*. *asper* venom. (PDF)
