# Supplementary material for: Muscle Tissue Damage Induced by the Venom of Bothrops asper: Identification of Early and Late Pathological Events through Proteomic Analysis
Source: PLoS Negl Trop Dis. 2016 Apr 1;10(4):e0004599. doi: 10.1371/journal.pntd.0004599 (PMC4818029; doi:10.1371/journal.pntd.0004599)
Supplement: S1 Table — (PDF) [file pntd.0004599.s001.pdf]

S1 Table. List of all proteins identified in wound exudates collected from mice at 1, 6 and 24 h after injection of *B. asper* venom.

Proteins identified		Accession Number	Molecular Mass	Quantitative Value		
				1h	6h	24h
1	Serum albumin OS=Mus musculus GN=Alb PE=1 SV=3	P07724	69 kDa	3,153	2,554	3,399
2	Cluster of Serotransferrin OS=Mus musculus GN=Tf PE=1 SV=1 (Q921I1)	Q921I1 [3]	77 kDa	1,191	1,057	1,229
3	Cluster of Isoform 3 of Titin OS=Mus musculus GN=Ttn (A2ASS6-3)	A2ASS6-3 [6]	619 kDa	630	1,026	229
4	Cluster of Hemoglobin subunit beta-2 OS=Mus musculus GN=Hbb-b2 PE=1 SV=2 (P02089)	P02089 [3]	16 kDa	746	924	1,330
5	Cluster of Complement C3 OS=Mus musculus GN=C3 PE=1 SV=3 (P01027)	P01027	186 kDa	692	672	883
6	Cluster of Serine protease inhibitor A3K OS=Mus musculus GN=Serpina3k PE=1 SV=2 (P07759)	P07759 [4]	47 kDa	821	711	1,292
7	Cluster of Alpha-2-macroglobulin OS=Mus musculus GN=Pzp PE=4 SV=1 (D3YW52)	D3YW52 [2]	167 kDa	782	527	712
8	Cluster of Alpha-1-antitrypsin 1-2 OS=Mus musculus GN=Serpina1b PE=1 SV=2 (P22599)	P22599 [4]	46 kDa	461	488	694
9	Creatine kinase M-type OS=Mus musculus GN=Ckm PE=1 SV=1	P07310	43 kDa	651	404	96
10	Cluster of Myosin-4 OS=Mus musculus GN=Myh4 PE=1 SV=1 (Q5SX39)	Q5SX39 [9]	223 kDa	43	620	529
11	Apolipoprotein A-I OS=Mus musculus GN=Apoa1 PE=1 SV=2	Q00623	31 kDa	292	202	183
12	Hemopexin OS=Mus musculus GN=Hpx PE=1 SV=2	Q91X72	51 kDa	265	223	356
13	Cluster of Alpha globin 1 OS=Mus musculus GN=haemaglobin alpha 1 PE=1 SV=1 (Q91VB8)	Q91VB8	15 kDa	233	267	436
14	Cluster of Phosphorylase OS=Mus musculus GN=Pygm PE=1 SV=1 (E9PUM3)	E9PUM3 [3]	88 kDa	300	294	47
15	Cluster of Fructose-bisphosphate aldolase OS=Mus musculus GN=Aldoa PE=2 SV=1 (A6ZI44)	A6ZI44 [3]	45 kDa	357	216	109
16	Murinoglobulin-1 OS=Mus musculus GN=Mug1 PE=1 SV=3	P28665	165 kDa	524	360	432
17	Fibronectin OS=Mus musculus GN=Fn1 PE=1 SV=4	P11276	273 kDa	275	194	291
18	Cluster of Ceruloplasmin OS=Mus musculus GN=Cp PE=4 SV=1 (G3X8Q5)	G3X8Q5 [2]	124 kDa	211	189	259

19	Cluster of Actin, alpha skeletal muscle OS=Mus musculus GN=Acta1 PE=1 SV=1 (P68134)	P68134 [7]	42 kDa	120	258	220
20	Cluster of Beta-enolase OS=Mus musculus GN=Eno3 PE=1 SV=3 (P21550)	P21550 [3]	47 kDa	216	147	73
21	Cluster of Complement factor H OS=Mus musculus GN=Cfh PE=1 SV=1 (E9Q8I0)	E9Q8I0	141 kDa	160	138	149
22	Cluster of Alpha-actinin-2 OS=Mus musculus GN=Actn2 PE=1 SV=2 (Q9JI91)	Q9JI91 [3]	104 kDa	190	298	59
23	Apolipoprotein A-IV OS=Mus musculus GN=Apoa4 PE=1 SV=3	P06728	45 kDa	120	167	216
24	Apolipoprotein B-100 OS=Mus musculus GN=Apob PE=1 SV=1	E9Q414	509 kDa	144	75	144
25	Vitamin D-binding protein OS=Mus musculus GN=Gc PE=1 SV=2	P21614	54 kDa	178	251	134
26	Cluster of Isoform M1 of Pyruvate kinase PKM OS=Mus musculus GN=Pkm (P52480-2)	P52480-2 [2]	58 kDa	195	169	81
27	Plasminogen OS=Mus musculus GN=Plg PE=1 SV=3	P20918	91 kDa	142	102	108
28	Cluster of Keratin, type II cytoskeletal 2 epidermal OS=Mus musculus GN=Krt2 PE=1 SV=1 (Q3TTY5)	Q3TTY5 [9]	71 kDa	94	132	90
29	Transthyretin OS=Mus musculus GN=Ttr PE=1 SV=1	P07309	16 kDa	144	80	127
30	Complement C4-B OS=Mus musculus GN=C4b PE=1 SV=3	P01029	193 kDa	134	76	109
31	Cluster of Inter alpha-trypsin inhibitor, heavy chain 4 OS=Mus musculus GN=Itih4 PE=1 SV=2 (A6X935)	A6X935 [2]	105 kDa	101	82	301
32	Cluster of Carboxylesterase 1C OS=Mus musculus GN=Ces1c PE=1 SV=4 (P23953)	P23953 [3]	61 kDa	200	168	144
33	Alpha-actinin-3 OS=Mus musculus GN=Actn3 PE=2 SV=1	O88990	103 kDa	161	206	55
34	Cluster of Uncharacterized protein OS=Mus musculus GN=Gm12117 PE=3 SV=1 (V9GXK0)	V9GXK0 [3]	16 kDa	274	124	37
35	Cluster of Apolipoprotein E OS=Mus musculus GN=Apoe PE=1 SV=2 (P08226)	P08226	36 kDa	124	142	133
36	Protein Fga OS=Mus musculus GN=Fga PE=4 SV=1	E9PV24	87 kDa	33	75	180
37	Prolow-density lipoprotein receptor-related protein 1 OS=Mus musculus GN=Lrp1 PE=1 SV=1	Q91ZX7	505 kDa	0	34	46
38	Cluster of Filamin-C OS=Mus musculus GN=Flnc PE=1 SV=3 (Q8VHX6)	Q8VHX6 [4]	291 kDa	111	122	17

39	Fibrinogen gamma chain OS=Mus musculus GN=Fgg PE=2 SV=1	Q8VCM7	49 kDa	50	118	145
40	Fatty acid synthase OS=Mus musculus GN=Fasn PE=1 SV=2	P19096	272 kDa	84	73	32
41	Cluster of Ig heavy chain Mem5 (Fragment) OS=Mus musculus PE=1 SV=1 (P84751)	P84751	25 kDa	80	93	127
42	Gelsolin OS=Mus musculus GN=Gsn PE=1 SV=3	P13020	86 kDa	76	76	92
43	Cluster of Protein Gm20547 OS=Mus musculus GN=Gm20547 PE=3 SV=1 (B8JJN0)	B8JJN0 [3]	142 kDa	72	74	73
44	Cluster of L-lactate dehydrogenase OS=Mus musculus GN=Ldha PE=1 SV=1 (G5E8N5)	G5E8N5 [2]	40 kDa	105	87	34
45	Myosin-binding protein C, fast-type OS=Mus musculus GN=Mybpc2 PE=1 SV=1	Q5XKE0	127 kDa	121	108	46
46	Cluster of Isoform 3 of Keratin, type I cytoskeletal 10 OS=Mus musculus GN=Krt10 (P02535-3)	P02535-3 [5]	50 kDa	80	70	63
47	Isoform 2 of Ig gamma-2B chain C region OS=Mus musculus GN=Igh-3	P01867-2	37 kDa	74	56	80
48	Antithrombin-III OS=Mus musculus GN=Serpinc1 PE=1 SV=1	P32261	52 kDa	55	70	57
49	Cluster of Kininogen-1 OS=Mus musculus GN=Kng1 PE=1 SV=1 (O08677)	O08677 [2]	73 kDa	117	83	76
50	Cluster of Sarcoplasmic/endoplasmic reticulum calcium ATPase 1 OS=Mus musculus GN=Atp2a1 PE=2 SV=1 (Q8R429)	Q8R429 [2]	109 kDa	80	77	0
51	Alpha-2-HS-glycoprotein OS=Mus musculus GN=Ahsg PE=1 SV=1	P29699	37 kDa	47	54	80
52	Murinoglobulin-2 OS=Mus musculus GN=Mug2 PE=2 SV=2	P28666	162 kDa	274	214	183
53	Cluster of Protein Tnxb OS=Mus musculus GN=Tnxb PE=4 SV=1 (O35452)	O35452 [2]	435 kDa	33	17	23
54	Triosephosphate isomerase OS=Mus musculus GN=Tpi1 PE=1 SV=4	P17751	32 kDa	98	96	27
55	Cluster of Fibrillin-1 OS=Mus musculus GN=Fbn1 PE=4 SV=1 (A2AQ53)	A2AQ53 [2]	312 kDa	0	22	0
56	Cluster of Phosphoglycerate kinase 1 OS=Mus musculus GN=Pgk1 PE=1 SV=4 (P09411)	P09411	45 kDa	58	47	15
57	Haptoglobin OS=Mus musculus GN=Hp PE=1 SV=1	Q61646	39 kDa	60	65	84
58	Carbonic anhydrase 3 OS=Mus musculus GN=Ca3 PE=1 SV=3	P16015	29 kDa	56	53	25
59	Inter-alpha trypsin inhibitor, heavy chain 2 OS=Mus musculus GN=Itih2 PE=1 SV=1	G3X977 (+1)	106 kDa	32	46	57

60	Ig gamma-1 chain C region, membrane-bound form OS=Mus musculus GN=Ighg1 PE=1 SV=2	P01869	43 kDa	73	68	57
61	Cluster of Inhibitor of carbonic anhydrase OS=Mus musculus GN=Ica PE=1 SV=1 (Q9DBD0)	Q9DBD0	77 kDa	58	64	62
62	Phosphoglycerate mutase 2 OS=Mus musculus GN=Pgam2 PE=1 SV=3	O70250	29 kDa	69	98	34
63	Cluster of Prothrombin OS=Mus musculus GN=F2 PE=1 SV=1 (P19221)	P19221	70 kDa	54	19	21
64	Myomesin 2 OS=Mus musculus GN=Myom2 PE=2 SV=1	Q14BI5	165 kDa	15	57	72
65	Parvalbumin alpha OS=Mus musculus GN=Pvalb PE=1 SV=3	P32848	12 kDa	58	38	37
66	Protein Agl OS=Mus musculus GN=Agl PE=4 SV=1	F8VPN4	174 kDa	60	37	0
67	Ig mu chain C region OS=Mus musculus GN=Ighm PE=1 SV=2	P01872	50 kDa	49	58	32
68	Ig kappa chain C region OS=Mus musculus PE=1 SV=1	P01837	12 kDa	42	21	32
69	Cluster of Heat shock cognate 71 kDa protein OS=Mus musculus GN=Hspa8 PE=1 SV=1 (P63017)	P63017 [4]	71 kDa	51	73	17
70	Fibrinogen beta chain OS=Mus musculus GN=Fgb PE=2 SV=1	Q8K0E8	55 kDa	13	66	108
71	Cluster of Heat shock protein HSP 90-beta OS=Mus musculus GN=Hsp90ab1 PE=1 SV=3 (P11499)	P11499 [2]	83 kDa	42	71	26
72	Afamin OS=Mus musculus GN=Afm PE=1 SV=2	O89020	69 kDa	34	38	35
73	Inter-alpha-trypsin inhibitor heavy chain H1 OS=Mus musculus GN=Itih1 PE=1 SV=2	Q61702	101 kDa	50	49	48
74	Malate dehydrogenase, mitochondrial OS=Mus musculus GN=Mdh2 PE=1 SV=3	P08249	36 kDa	65	63	14
75	Carbonic anhydrase 2 OS=Mus musculus GN=Ca2 PE=1 SV=4	P00920	29 kDa	55	39	33
76	Fibrillin-2 OS=Mus musculus GN=Fbn2 PE=1 SV=2	Q61555	314 kDa	1	22	0
77	Aconitate hydratase, mitochondrial OS=Mus musculus GN=Aco2 PE=1 SV=1	Q99KI0	85 kDa	48	99	10
78	Cluster of Alpha-1-acid glycoprotein 1 OS=Mus musculus GN=Orm1 PE=1 SV=1 (Q60590)	Q60590 [2]	24 kDa	14	10	59
79	Beta-2-glycoprotein 1 OS=Mus musculus GN=Apoh PE=1 SV=1	Q01339	39 kDa	33	37	23

80	Complement factor I OS=Mus musculus GN=Cfi PE=1 SV=3	Q61129	67 kDa	19	37	26
81	ATP-dependent 6-phosphofructokinase, muscle type OS=Mus musculus GN=Pfkm PE=1 SV=3	P47857	85 kDa	68	27	0
82	Cluster of Obscurin OS=Mus musculus GN=Obscn PE=2 SV=2 (A2AAJ9)	A2AAJ9 [4]	966 kDa	18	17	0
83	Cluster of Phosphoglucomutase-1 OS=Mus musculus GN=Pgm1 PE=1 SV=4 (Q9D0F9)	Q9D0F9 [2]	61 kDa	68	34	69
84	Cluster of Desmin OS=Mus musculus GN=Des PE=1 SV=3 (P31001)	P31001 [2]	53 kDa	29	40	27
85	Cluster of Nesprin-1 OS=Mus musculus GN=Syne1 PE=1 SV=2 (Q6ZWR6)	Q6ZWR6	1010 kDa	0	45	0
86	Cluster of Glycosylphosphatidylinositol specific phospholipase D1 OS=Mus musculus GN=Gpld1 PE=2 SV=1 (Q8VCU2)	Q8VCU2	94 kDa	42	41	21
87	Peroxiredoxin-2 OS=Mus musculus GN=Prdx2 PE=1 SV=3	Q61171	22 kDa	43	38	62
88	Alpha-1B-glycoprotein OS=Mus musculus GN=A1bg PE=1 SV=1	Q19LI2	57 kDa	10	30	50
89	Cluster of Basement membrane-specific heparan sulfate proteoglycan core protein OS=Mus musculus GN=Hspg2 PE=4 SV=1 (B1B0C7)	B1B0C7 [2]	469 kDa	83	56	0
90	Plasma protease C1 inhibitor OS=Mus musculus GN=Serping1 PE=1 SV=3	P97290	56 kDa	31	35	34
91	Cluster of Alpha-2-antiplasmin OS=Mus musculus GN=Serpinf2 PE=1 SV=1 (Q61247)	Q61247	55 kDa	34	26	15
92	Cluster of Leukemia inhibitory factor receptor OS=Mus musculus GN=Lifr PE=1 SV=1 (P42703)	P42703 [2]	123 kDa	33	25	42
93	Apolipoprotein A-II OS=Mus musculus GN=Apoa2 PE=1 SV=2	P09813	11 kDa	46	67	32
94	Serum amyloid P-component OS=Mus musculus GN=Apcs PE=1 SV=2	P12246	26 kDa	28	28	65
95	Histidine-rich glycoprotein OS=Mus musculus GN=Hrg PE=1 SV=2	Q9ESB3	59 kDa	33	27	34
96	Histone H4 OS=Mus musculus GN=Hist1h4a PE=1 SV=2	P62806	11 kDa	56	48	47
97	Cluster of Tubulin beta-5 chain OS=Mus musculus GN=Tubb5 PE=1 SV=1 (P99024)	P99024 [3]	50 kDa	49	43	26
98	Retinol-binding protein 4 OS=Mus musculus GN=Rbp4 PE=3 SV=1	H7BWY6	28 kDa	39	33	31

99	Cluster of H-2 class I histocompatibility antigen, Q10 alpha chain OS=Mus musculus GN=H2-Q10 PE=1 SV=3 (P01898)	P01898 [5]	37 kDa	18	24	14
100	Cluster of 14-3-3 protein epsilon OS=Mus musculus GN=Ywhae PE=1 SV=1 (P62259)	P62259	29 kDa	42	44	33
101	Cluster of Plectin OS=Mus musculus GN=Plec PE=1 SV=3 (Q9QXS1)	Q9QXS1 [4]	534 kDa	16	18	0
102	Cluster of von Willebrand factor (Fragment) OS=Mus musculus GN=Vwf PE=4 SV=1 (S4R195)	S4R195 [2]	42 kDa	65	90	46
103	Cluster of Glycerol-3-phosphate dehydrogenase [NAD(+)], cytoplasmic OS=Mus musculus GN=Gpd1 PE=1 SV=3 (P13707)	P13707	38 kDa	48	24	34
104	Cluster of Glucose-6-phosphate isomerase OS=Mus musculus GN=Gpi PE=1 SV=4 (P06745)	P06745	63 kDa	31	52	17
105	Cluster of Tubulin alpha-1C chain OS=Mus musculus GN=Tuba1c PE=1 SV=1 (P68373)	P68373 [3]	50 kDa	31	27	17
106	Cluster of Nucleoside diphosphate kinase OS=Mus musculus GN=Gm20390 PE=3 SV=1 (E9PZF0)	E9PZF0 [2]	30 kDa	16	19	18
107	Isoform 3 of Sulfhydryl oxidase 1 OS=Mus musculus GN=Qsox1	Q8BND5-3	63 kDa	50	33	35
108	Inter-alpha-trypsin inhibitor heavy chain H3 OS=Mus musculus GN=Itih3 PE=1 SV=3	Q61704	99 kDa	10	13	47
109	Vitronectin OS=Mus musculus GN=Vtn PE=1 SV=2	P29788	55 kDa	19	13	10
110	Transitional endoplasmic reticulum ATPase OS=Mus musculus GN=Vcp PE=1 SV=4	Q01853	89 kDa	25	35	15
111	Cluster of Myosin regulatory light chain 2, skeletal muscle isoform OS=Mus musculus GN=Mylpf PE=1 SV=3 (P97457)	P97457	19 kDa	46	30	74
112	Cluster of Peptidyl-prolyl cis-trans isomerase A OS=Mus musculus GN=Ppia PE=1 SV=2 (P17742)	P17742	18 kDa	22	67	24
113	Cluster of Filamin, alpha OS=Mus musculus GN=Flna PE=1 SV=1 (B7FAU9)	B7FAU9	280 kDa	15	24	18
114	Cluster of Glutathione S-transferase Mu 2 OS=Mus musculus GN=Gstm2 PE=1 SV=2 (P15626)	P15626 [3]	26 kDa	13	22	13
115	Cluster of Epidermal growth factor receptor OS=Mus musculus GN=Egfr PE=1 SV=1 (Q01279)	Q01279	135 kDa	17	24	92

116	Histone H3.1 OS=Mus musculus GN=Hist1h3a PE=1 SV=2	P68433	15 kDa	19	12	11
117	Cluster of Clathrin heavy chain 1 OS=Mus musculus GN=Cltc PE=1 SV=3 (Q68FD5)	Q68FD5	192 kDa	17	18	92
118	Elongation factor 2 OS=Mus musculus GN=Eef2 PE=1 SV=2	P58252	95 kDa	11	36	46
119	Malate dehydrogenase, cytoplasmic OS=Mus musculus GN=Mdh1 PE=1 SV=3	P14152	37 kDa	43	34	80
120	Cluster of Histone H2B type 1-M OS=Mus musculus GN=Hist1h2bm PE=1 SV=2 (P10854)	P10854 [3]	14 kDa	46	21	30
121	Talin-1 OS=Mus musculus GN=Tln1 PE=1 SV=2	P26039	270 kDa	83	33	33
122	Cluster of Cytochrome c, somatic OS=Mus musculus GN=Cycs PE=1 SV=2 (P62897)	P62897	12 kDa	27	18	16
123	Cluster of Protein DJ-1 OS=Mus musculus GN=Park7 PE=1 SV=1 (Q99LX0)	Q99LX0	20 kDa	17	15	10
124	Cluster of Protein Mybpc1 OS=Mus musculus GN=Mybpc1 PE=2 SV=1 (Q6P6L5)	Q6P6L5	126 kDa	17	28	0
125	Carboxypeptidase N subunit 2 OS=Mus musculus GN=Cpn2 PE=1 SV=2	Q9DBB9	60 kDa	23	41	11
126	Cluster of Protein Ighg2c OS=Mus musculus GN=Ighg2c PE=4 SV=1 (F6TQW2)	F6TQW2	44 kDa	27	20	49
127	Cluster of Creatine kinase S-type, mitochondrial OS=Mus musculus GN=Ckmt2 PE=1 SV=1 (Q6P8J7)	Q6P8J7	47 kDa	17	28	11
128	Carbonic anhydrase 1 OS=Mus musculus GN=Ca1 PE=2 SV=4	P13634	28 kDa	38	19	22
129	Cluster of Glutathione peroxidase 3 OS=Mus musculus GN=Gpx3 PE=2 SV=2 (P46412)	P46412	25 kDa	30	22	27
130	Cluster of Proteoglycan 4 OS=Mus musculus GN=Prg4 PE=4 SV=1 (E9QQ17)	E9QQ17 [4]	111 kDa	19	11	13
131	Isoform 2 of Myomesin-1 OS=Mus musculus GN=Myom1	Q62234-2	175 kDa	65	16	11
132	Vinculin OS=Mus musculus GN=Vcl PE=1 SV=4	Q64727	117 kDa	17	26	19
133	Cluster of Ubiquitin-40S ribosomal protein S27a OS=Mus musculus GN=Rps27a PE=1 SV=2 (P62983)	P62983 [2]	18 kDa	74	15	26
134	Cluster of Cytoplasmic dynein 1 heavy chain 1 OS=Mus musculus GN=Dync1h1 PE=1 SV=2 (Q9JHU4)	Q9JHU4	532 kDa	12	45	11
135	Collagen alpha-1(XIV) chain OS=Mus musculus GN=Col14a1 PE=2 SV=1	B7ZNH7	193 kDa	12	16	29

136	Plasma kallikrein OS=Mus musculus GN=Klkb1 PE=1 SV=2	P26262	71 kDa	23	24	92
137	Fetuin-B OS=Mus musculus GN=Fetub PE=1 SV=1	Q9QXC1	43 kDa	18	24	11
138	Peroxiredoxin-1 OS=Mus musculus GN=Prdx1 PE=1 SV=1	P35700	22 kDa	27	27	19
139	Cluster of Clusterin OS=Mus musculus GN=Clu PE=1 SV=1 (Q06890)	Q06890	52 kDa	16	16	17
140	Complement component C8 gamma chain OS=Mus musculus GN=C8g PE=1 SV=1	Q8VCG4	23 kDa	31	28	32
141	Cluster of Four and a half LIM domains 1, isoform CRA_b OS=Mus musculus GN=Fhl1 PE=1 SV=1 (A2AEX8)	A2AEX8 [2]	34 kDa	32	21	0
142	Fatty acid-binding protein, adipocyte OS=Mus musculus GN=Fabp4 PE=1 SV=3	P04117	15 kDa	24	19	19
143	Thrombospondin-1 OS=Mus musculus GN=Thbs1 PE=1 SV=1	P35441	130 kDa	65	16	11
144	Corticosteroid-binding globulin OS=Mus musculus GN=Serpina6 PE=1 SV=1	Q06770	45 kDa	30	90	10
145	Maltase-glucoamylase OS=Mus musculus GN=Mgam PE=2 SV=1	B5THE2	209 kDa	24	17	14
146	Aspartate aminotransferase, mitochondrial OS=Mus musculus GN=Got2 PE=1 SV=1	P05202	47 kDa	26	25	46
147	Elongation factor 1-gamma OS=Mus musculus GN=Eef1g PE=1 SV=3	Q9D8N0	50 kDa	16	22	69
148	Cluster of Ig heavy chain V region AC38 205.12 OS=Mus musculus PE=1 SV=1 (P06330)	P06330 [3]	13 kDa	13	16	13
149	Protein S100-A9 OS=Mus musculus GN=S100a9 PE=1 SV=3	P31725	13 kDa	1	56	22
150	Cluster of Ras-related protein Rab-14 OS=Mus musculus GN=Rab14 PE=3 SV=1 (A2AL34)	A2AL34 [7]	17 kDa	93	56	69
151	Cluster of Myosin light chain 1/3, skeletal muscle isoform OS=Mus musculus GN=Myl1 PE=1 SV=2 (P05977)	P05977 [2]	21 kDa	10	26	59
152	Ryanodine receptor 1 OS=Mus musculus GN=Ryr1 PE=1 SV=1	E9PZQ0	565 kDa	19	45	0
153	Lumican OS=Mus musculus GN=Lum PE=1 SV=2	P51885	38 kDa	11	11	17
154	Protein Krt78 OS=Mus musculus GN=Krt78 PE=4 SV=1	E9Q0F0	112 kDa	16	17	11
155	Troponin C, skeletal muscle OS=Mus musculus GN=Tnnc2 PE=1 SV=2	P20801	18 kDa	13	29	32
156	ATP-binding cassette sub-family A member 8-B OS=Mus musculus GN=Abca8b PE=2 SV=2	Q8K440	183 kDa	93	45	57

157	Protein AMBP OS=Mus musculus GN=Ambp PE=2 SV=2	Q07456	39 kDa	19	15	30
158	Cluster of Ig kappa chain V-V region HP 123E6 OS=Mus musculus PE=1 SV=1 (P01646)	P01646	12 kDa	16	11	19
159	Myoglobin OS=Mus musculus GN=Mb PE=1 SV=3	P04247	17 kDa	13	19	11
160	Cluster of Complement component C9 OS=Mus musculus GN=C9 PE=1 SV=2 (P06683)	P06683	62 kDa	56	45	23
161	Myosin-9 OS=Mus musculus GN=Myh9 PE=1 SV=4	Q8VDD5	226 kDa	65	10	31
162	Troponin I, fast skeletal muscle OS=Mus musculus GN=Tnni2 PE=1 SV=2	P13412	21 kDa	46	12	16
163	Complement component C8 alpha chain OS=Mus musculus GN=C8a PE=2 SV=1	Q8K182	66 kDa	16	16	57
164	Cluster of Citrate synthase, mitochondrial OS=Mus musculus GN=Cs PE=1 SV=1 (Q9CZU6)	Q9CZU6 [2]	52 kDa	56	17	11
165	Transketolase OS=Mus musculus GN=Tkt PE=1 SV=1	P40142	68 kDa	37	10	15
166	Myeloid bactenecin (F1) OS=Mus musculus GN=Ngp PE=2 SV=1	O08692	19 kDa	0	67	50
167	14-3-3 protein sigma OS=Mus musculus GN=Sfn PE=1 SV=2	O70456	28 kDa	37	28	35
168	Kelch-like protein 41 OS=Mus musculus GN=Klhl41 PE=1 SV=1	A2AUC9	68 kDa	65	78	0
169	Collagen alpha-2(I) chain OS=Mus musculus GN=Col1a2 PE=2 SV=2	Q01149	130 kDa	16	22	57
170	Ig kappa chain V-V region K2 (Fragment) OS=Mus musculus PE=1 SV=1	P01635	13 kDa	83	12	30
171	Xanthine dehydrogenase/oxidase OS=Mus musculus GN=Xdh PE=1 SV=5	Q00519	147 kDa	1	22	34
172	Alpha-2-macroglobulin-P OS=Mus musculus GN=A2mp PE=2 SV=2	Q6GQT1	164 kDa	26	34	57
173	Cluster of Isoform Short of 14-3-3 protein beta/alpha OS=Mus musculus GN=Ywhab (Q9CQV8-2)	Q9CQV8-2 [2]	28 kDa	46	52	48
174	Cluster of Laminin subunit gamma-1 OS=Mus musculus GN=Lamc1 PE=1 SV=2 (P02468)	P02468 [2]	177 kDa	1	56	46
175	Serum amyloid A-4 protein OS=Mus musculus GN=Saa4 PE=1 SV=2	P31532	15 kDa	13	22	11
176	Elongation factor 1-alpha 2 OS=Mus musculus GN=Eef1a2 PE=1 SV=1	P62631	50 kDa	43	31	23

177	Cluster of Flavin reductase (NADPH) OS=Mus musculus GN=Blvrb PE=2 SV=3 (Q923D2)	Q923D2	22 kDa	74	15	22
178	Zinc-alpha-2-glycoprotein OS=Mus musculus GN=Azgp1 PE=1 SV=2	Q64726	35 kDa	93	16	16
179	Heparin cofactor 2 OS=Mus musculus GN=Serpind1 PE=1 SV=1	P49182	54 kDa	28	67	23
180	Cluster of Bisphosphoglycerate mutase OS=Mus musculus GN=Bpgm PE=2 SV=2 (P15327)	P15327 [2]	30 kDa	93	15	14
181	Profilin-1 OS=Mus musculus GN=Pfn1 PE=1 SV=2	P62962	15 kDa	11	18	26
182	Cluster of Angiotensinogen OS=Mus musculus GN=Agt PE=2 SV=1 (Q3UTR7)	Q3UTR7	53 kDa	83	19	22
183	Cluster of Ubiquitin-like modifier-activating enzyme 1 OS=Mus musculus GN=Uba1 PE=1 SV=1 (Q02053)	Q02053	118 kDa	12	19	46
184	Cluster of 14-3-3 protein zeta/delta OS=Mus musculus GN=Ywhaz PE=1 SV=1 (P63101)	P63101	28 kDa	20	41	38
185	Cluster of Histone H2A OS=Mus musculus GN=Hist1h2aa PE=2 SV=1 (Q8CGP4)	Q8CGP4 [2]	14 kDa	28	20	34
186	Cluster of Serum paraoxonase/arylesterase 1 OS=Mus musculus GN=Pon1 PE=1 SV=2 (P52430)	P52430	40 kDa	20	16	10
187	Phosphatidylethanolamine-binding protein 1 OS=Mus musculus GN=Pebp1 PE=1 SV=3	P70296	21 kDa	14	17	57
188	Beta-2-microglobulin OS=Mus musculus GN=B2m PE=1 SV=2	P01887	14 kDa	74	11	17
189	Cluster of MCG48959 OS=Mus musculus GN=Prdx6b PE=2 SV=1 (Q8BG37)	Q8BG37 [2]	25 kDa	74	18	13
190	Cluster of Peroxiredoxin-5, mitochondrial OS=Mus musculus GN=Prdx5 PE=1 SV=2 (P99029)	P99029 [2]	22 kDa	83	16	17
191	Spectrin beta chain, non-erythrocytic 1 OS=Mus musculus GN=Sptbn1 PE=1 SV=2	Q62261	274 kDa	19	22	46
192	Cluster of Glutathione S-transferase P 1 OS=Mus musculus GN=Gstp1 PE=1 SV=2 (P19157)	P19157	24 kDa	16	15	80
193	Mannose-binding protein A OS=Mus musculus GN=Mbl1 PE=2 SV=1	P39039	25 kDa	29	11	11
194	Coagulation factor XII OS=Mus musculus GN=F12 PE=2 SV=2	Q80YC5	66 kDa	65	22	69
195	Apolipoprotein M OS=Mus musculus GN=Apom PE=1 SV=1	Q9Z1R3	21 kDa	10	90	34

196	Cluster of Transferrin receptor protein 1 OS=Mus musculus GN=Tfrc PE=1 SV=1 (Q62351)	Q62351	86 kDa	12	15	23
197	Cluster of Aspartate aminotransferase, cytoplasmic OS=Mus musculus GN=Got1 PE=1 SV=3 (P05201)	P05201	46 kDa	11	18	11
198	Cluster of Protein Col6a3 OS=Mus musculus GN=Col6a3 PE=1 SV=2 (E9PWQ3)	E9PWQ3	354 kDa	56	45	0
199	Collagen alpha-1(I) chain OS=Mus musculus GN=Col1a1 PE=1 SV=4	P11087	138 kDa	15	13	57
200	Apolipoprotein D OS=Mus musculus GN=Apod PE=2 SV=1	P51910	22 kDa	19	90	13
201	Cluster of Spectrin alpha chain, non-erythrocytic 1 OS=Mus musculus GN=Sptan1 PE=1 SV=4 (P16546)	P16546 [2]	285 kDa	19	45	34
202	LIM domain-binding protein 3 OS=Mus musculus GN=Ldb3 PE=4 SV=1	E9PYJ9 (+2)	72 kDa	16	11	0
203	Leucine-rich HEV glycoprotein OS=Mus musculus GN=Lrg1 PE=2 SV=1	Q91XL1	37 kDa	65	12	27
204	Cluster of Major urinary protein 8 OS=Mus musculus GN=Mup10 PE=2 SV=1 (A2BIN1)	A2BIN1	21 kDa	56	90	14
205	Isoform 2 of Ig gamma-3 chain C region OS=Mus musculus	P03987-2	36 kDa	74	78	80
206	Glutathione peroxidase 1 OS=Mus musculus GN=Gpx1 PE=1 SV=2	P11352	22 kDa	83	13	14
207	Fatty acid-binding protein, heart OS=Mus musculus GN=Fabp3 PE=1 SV=5	P11404	15 kDa	15	78	0
208	Cluster of ADP-ribosylation factor 4 OS=Mus musculus GN=Arf4 PE=1 SV=2 (P61750)	P61750 [2]	20 kDa	10	90	92
209	Thrombospondin-4 OS=Mus musculus GN=Thbs4 PE=1 SV=1	Q9Z1T2	106 kDa	65	34	46
210	Insulin-like growth factor-binding protein complex acid labile subunit OS=Mus musculus GN=Igfals PE=2 SV=1	P70389	67 kDa	10	15	11
211	Isoform 4 of Periostin OS=Mus musculus GN=Postn	Q62009-4	87 kDa	1	16	69
212	Complement component C8 beta chain OS=Mus musculus GN=C8b PE=1 SV=1	Q8BH35	66 kDa	56	90	23
213	Cluster of Proteasome subunit alpha type-7 OS=Mus musculus GN=Psma7 PE=1 SV=1 (Q9Z2U0)	Q9Z2U0	28 kDa	65	13	14
214	Cluster of Elongation factor 1-alpha 1 OS=Mus musculus GN=Eef1a1 PE=1 SV=3 (P10126)	P10126	50 kDa	31	36	29

215	Pigment epithelium-derived factor OS=Mus musculus GN=Serpinf1 PE=1 SV=2	P97298	46 kDa	28	15	80
216	C-type lectin domain family 3, member b OS=Mus musculus GN=Clec3b PE=2 SV=1	Q8CFZ6	22 kDa	65	67	80
217	Carboxypeptidase N catalytic chain OS=Mus musculus GN=Cpn1 PE=2 SV=1	Q9JJN5	52 kDa	12	90	92
218	UTP--glucose-1-phosphate uridylyltransferase OS=Mus musculus GN=Ugp2 PE=2 SV=3	Q91ZJ5	57 kDa	74	78	11
219	Cluster of Protein disulfide-isomerase OS=Mus musculus GN=P4hb PE=1 SV=2 (P09103)	P09103	57 kDa	28	17	69
220	Nidogen-2 OS=Mus musculus GN=Nid2 PE=1 SV=2	O88322	154 kDa	19	11	0
221	Spectrin alpha chain, erythrocytic 1 OS=Mus musculus GN=Spta1 PE=2 SV=3	P08032	280 kDa	0	56	34
222	Collagen alpha-1(III) chain OS=Mus musculus GN=Col3a1 PE=1 SV=4	P08121	139 kDa	12	45	23
223	C-reactive protein OS=Mus musculus GN=Crp PE=2 SV=2	P14847	25 kDa	65	10	19
224	Probable C->U-editing enzyme APOBEC-2 OS=Mus musculus GN=Apobec2 PE=1 SV=1	Q9WV35	26 kDa	83	18	92
225	Cluster of Collagen alpha-2(IV) chain OS=Mus musculus GN=Col4a2 PE=2 SV=4 (P08122)	P08122	167 kDa	37	11	0
226	Isoform Cytoplasmic of Fumarate hydratase, mitochondrial OS=Mus musculus GN=Fh	P97807-2	50 kDa	46	15	69
227	Cluster of Selenoprotein P OS=Mus musculus GN=Sepp1 PE=2 SV=3 (P70274)	P70274	43 kDa	1	34	11
228	Alpha-crystallin B chain OS=Mus musculus GN=Cryab PE=1 SV=2	P23927	20 kDa	18	34	0
229	Nucleolar pre-ribosomal-associated protein 1 OS=Mus musculus GN=Urb1 PE=4 SV=1	E9PU96 (+1)	255 kDa	19	11	11
230	Cluster of Adenylate kinase isoenzyme 1 OS=Mus musculus GN=Ak1 PE=1 SV=1 (Q9R0Y5)	Q9R0Y5 [2]	22 kDa	20	78	23
231	Protein 9530053A07Rik OS=Mus musculus GN=9530053A07Rik PE=4 SV=1	E9PVG8	280 kDa	19	34	11
232	Complement C5 OS=Mus musculus GN=C5 PE=1 SV=2	P06684	189 kDa	28	22	0
233	Cluster of GTP-binding nuclear protein Ran, testis-specific isoform OS=Mus musculus GN=Rasl2-9 PE=2 SV=1 (Q61820)	Q61820 [2]	24 kDa	11	34	80
234	Sarcalumenin OS=Mus musculus GN=Srl PE=1 SV=1	Q7TQ48	99 kDa	10	45	0
235	Rho GDP-dissociation inhibitor 1 OS=Mus musculus GN=Arhgdia PE=1 SV=3	Q99PT1	23 kDa	14	11	11

236	Cluster of Ig kappa chain V-V region L6 (Fragment) OS=Mus musculus PE=4 SV=1 (P01638)	P01638	13 kDa	46	67	10
237	Superoxide dismutase [Cu-Zn] OS=Mus musculus GN=Sod1 PE=1 SV=2	P08228	16 kDa	28	10	57
238	mitochondrial OS=Mus musculus GN=Idh2 PE=1 SV=3	P54071	51 kDa	19	78	0
239	Cluster of Ig kappa chain V-II region 26-10 OS=Mus musculus PE=1 SV=1 (P01631)	P01631	12 kDa	83	90	80
240	Cluster of Ig heavy chain V region MOPC 173 OS=Mus musculus PE=1 SV=1 (P01812)	P01812 [3]	13 kDa	74	45	11
241	Complement C1q subcomponent subunit A OS=Mus musculus GN=C1qa PE=1 SV=2	P98086	26 kDa	74	78	10
242	Oxidation resistance protein 1 OS=Mus musculus GN=C7 PE=4 SV=2	D3YXF5	93 kDa	37	78	11
243	Fatty acid-binding protein, epidermal OS=Mus musculus GN=Fabp5 PE=1 SV=3	Q05816	15 kDa	19	19	15
244	Cluster of L-lactate dehydrogenase B chain OS=Mus musculus GN=Ldhb PE=1 SV=2 (P16125)	P16125	37 kDa	65	18	80
245	Cluster of Ig heavy chain V region B1-8/186-2 OS=Mus musculus GN=Ighv1-72 PE=1 SV=1 (P01751)	P01751 [2]	15 kDa	37	34	0
246	Cluster of Moesin OS=Mus musculus GN=Msn PE=1 SV=3 (P26041)	P26041	68 kDa	0	11	80
247	Cluster of Isoform 2 of Myc box-dependent-interacting protein 1 OS=Mus musculus GN=Bin1 (O08539-2)	O08539-2 [2]	48 kDa	83	22	0
248	Mannose-binding protein C OS=Mus musculus GN=Mbl2 PE=2 SV=2	P41317	26 kDa	11	90	92
249	Cluster of Protein Z-dependent protease inhibitor OS=Mus musculus GN=Serpina10 PE=1 SV=1 (Q8R121)	Q8R121	52 kDa	1	34	34
250	Cluster of C4b-binding protein OS=Mus musculus GN=C4bpa PE=1 SV=3 (P08607)	P08607	52 kDa	65	34	23
251	Nidogen-1 OS=Mus musculus GN=Nid1 PE=1 SV=2	P10493	137 kDa	19	56	0
252	Cluster of Fibulin-1 OS=Mus musculus GN=Fbln1 PE=1 SV=2 (Q08879)	Q08879	78 kDa	0	34	0
253	Cluster of Tropomyosin alpha-3 chain OS=Mus musculus GN=Tpm3 PE=3 SV=1 (E9Q5J9)	E9Q5J9 [5]	33 kDa	1	67	23
254	Cluster of Complement C1s-A subcomponent OS=Mus musculus GN=C1sa PE=2 SV=2 (Q8CG14)	Q8CG14 [3]	77 kDa	74	11	23

255	Transgelin-2 OS=Mus musculus GN=Tagln2 PE=1 SV=4	Q9WVA4	22 kDa	56	10	16
256	Ig kappa chain V19-17 OS=Mus musculus GN=Igk-V19-17 PE=1 SV=1	P01633	16 kDa	19	45	27
257	Proteasome subunit alpha type-6 OS=Mus musculus GN=Psma6 PE=1 SV=1	Q9QUM9	27 kDa	37	10	11
258	Protein Sptbn2 OS=Mus musculus GN=Sptbn2 PE=1 SV=1	Q68FG2	271 kDa	0	0	23
259	Proteasome subunit beta type-5 OS=Mus musculus GN=Psmb5 PE=1 SV=3	O55234	29 kDa	15	90	34
260	Ig alpha chain C region OS=Mus musculus PE=1 SV=1	P01878	37 kDa	12	45	92
261	Cluster of Purine nucleoside phosphorylase OS=Mus musculus GN=Pnp PE=1 SV=2 (P23492)	P23492 [2]	32 kDa	37	12	92
262	Succinyl-CoA ligase [ADP-forming] subunit beta, mitochondrial OS=Mus musculus GN=Sucla2 PE=1 SV=2	Q9Z2I9	50 kDa	0	45	0
263	14-3-3 protein gamma OS=Mus musculus GN=Ywhag PE=1 SV=2	P61982	28 kDa	50	22	33
264	Myotilin OS=Mus musculus GN=Myot PE=1 SV=1	Q9JIF9	55 kDa	46	78	0
265	Complement C1q subcomponent subunit B OS=Mus musculus GN=C1qb PE=1 SV=2	P14106	27 kDa	10	67	80
266	Prelamin-A/C OS=Mus musculus GN=Lmna PE=1 SV=2	P48678	74 kDa	1	78	57
267	Isocitrate dehydrogenase [NADP] cytoplasmic OS=Mus musculus GN=Idh1 PE=1 SV=2	O88844	47 kDa	19	10	23
268	ATP-citrate synthase OS=Mus musculus GN=Acly PE=1 SV=1	Q91V92	120 kDa	65	90	23
269	Cluster of Isoform 3 of Glyoxalase domain-containing protein 4 OS=Mus musculus GN=Glod4 (Q9CPV4-3)	Q9CPV4-3	31 kDa	74	78	57
270	Proteasome subunit alpha type-1 OS=Mus musculus GN=Psma1 PE=1 SV=1	Q9R1P4	30 kDa	46	67	57
271	Early endosome antigen 1 OS=Mus musculus GN=Eea1 PE=1 SV=2	Q8BL66	161 kDa	0	22	0
272	Myosin-10 OS=Mus musculus GN=Myh10 PE=1 SV=1	Q3UH59	233 kDa	1	34	23
273	Neutrophil gelatinase-associated lipocalin OS=Mus musculus GN=Lcn2 PE=1 SV=1	P11672	23 kDa	0	34	11
274	Proteasome subunit beta type-4 OS=Mus musculus GN=Psmb4 PE=1 SV=1	P99026	29 kDa	56	67	80

275	Rho GDP-dissociation inhibitor 2 OS=Mus musculus GN=Arhgdib PE=1 SV=3	Q61599	23 kDa	0	10	11
276	Galectin OS=Mus musculus GN=Lgals7 PE=2 SV=1	Q9CRB1	15 kDa	19	45	69
277	Cluster of Protein C6 OS=Mus musculus GN=C6 PE=4 SV=1 (E9Q6D8)	E9Q6D8 [2]	104 kDa	0	22	11
278	40S ribosomal protein S16 OS=Mus musculus GN=Rps16 PE=2 SV=4	P14131	16 kDa	19	34	34
279	3-ketoacyl-CoA thiolase, mitochondrial OS=Mus musculus GN=Acaa2 PE=1 SV=3	Q8BWT1	42 kDa	28	90	11
280	Proteasome subunit alpha type-4 OS=Mus musculus GN=Psma4 PE=1 SV=1	Q9R1P0	29 kDa	56	11	92
281	Cluster of Aldose reductase OS=Mus musculus GN=Akr1b1 PE=1 SV=3 (P45376)	P45376	36 kDa	56	34	23
282	Protein NDRG2 OS=Mus musculus GN=Ndrg2 PE=1 SV=1	Q9QYG0 (+1)	41 kDa	13	45	0
283	Alcohol dehydrogenase class-3 OS=Mus musculus GN=Adh5 PE=1 SV=3	P28474	40 kDa	1	34	11
284	Cluster of Proteasome subunit alpha type-5 OS=Mus musculus GN=Psma5 PE=1 SV=1 (Q9Z2U1)	Q9Z2U1	26 kDa	46	90	92
285	Cholinesterase OS=Mus musculus GN=Bche PE=2 SV=2	Q03311	68 kDa	1	0	34
286	Cofilin-2 OS=Mus musculus GN=Cfl2 PE=1 SV=1	P45591	19 kDa	17	78	46
287	Succinate dehydrogenase [ubiquinone] iron-sulfur subunit, mitochondrial OS=Mus musculus GN=Sdhb PE=1 SV=1	Q9CQA3	32 kDa	1	78	92
288	Cluster of Importin-5 OS=Mus musculus GN=Ipo5 PE=1 SV=3 (Q8BKC5)	Q8BKC5	124 kDa	1	45	0
289	Cluster of Keratin, type I cuticular Ha5 OS=Mus musculus GN=Krt35 PE=2 SV=1 (Q497I4)	Q497I4	51 kDa	65	67	46
290	Cluster of Cullin-associated NEDD8-dissociated protein 1 OS=Mus musculus GN=Cand1 PE=2 SV=2 (Q6ZQ38)	Q6ZQ38	136 kDa	0	34	23
291	Enoyl-CoA delta isomerase 1, mitochondrial OS=Mus musculus GN=Eci1 PE=1 SV=2	P42125	32 kDa	56	56	46
292	Cluster of Eukaryotic translation initiation factor 5A-1 OS=Mus musculus GN=Eif5a PE=1 SV=2 (P63242)	P63242 [2]	17 kDa	46	22	46
293	Cluster of Isoform 3 of 2-oxoglutarate dehydrogenase, mitochondrial OS=Mus musculus GN=Ogdh (Q60597-3)	Q60597-3	118 kDa	1	34	0

294	Cluster of Isoform 2 of Cytosol aminopeptidase OS=Mus musculus GN=Lap3 (Q9CPY7-2)	Q9CPY7-2	53 kDa	0	90	23
295	Peptidyl-prolyl cis-trans isomerase C OS=Mus musculus GN=Ppic PE=1 SV=1	P30412	23 kDa	74	90	92
296	Complement C1q subcomponent subunit C OS=Mus musculus GN=C1qc PE=2 SV=2	Q02105	26 kDa	93	67	46
297	Lactoylglutathione lyase OS=Mus musculus GN=Glo1 PE=1 SV=3	Q9CPU0	21 kDa	46	11	57
298	Cluster of Collagen alpha-1(XVIII) chain OS=Mus musculus GN=Col18a1 PE=4 SV=1 (E9QPX1)	E9QPX1 [2]	182 kDa	74	22	0
299	Adenosylhomocysteinase OS=Mus musculus GN=Ahcy PE=1 SV=3	P50247	48 kDa	0	19	57
300	Cluster of Adiponectin OS=Mus musculus GN=Adipoq PE=1 SV=2 (Q60994)	Q60994	27 kDa	93	12	69
301	Electron transfer flavoprotein subunit alpha, mitochondrial OS=Mus musculus GN=Etfa PE=1 SV=2	Q99LC5	35 kDa	14	78	0
302	Coagulation factor X OS=Mus musculus GN=F10 PE=1 SV=1	O88947	54 kDa	28	0	11
303	60S ribosomal protein L12 OS=Mus musculus GN=Rpl12 PE=1 SV=2	P35979	18 kDa	28	11	46
304	Electron transfer flavoprotein subunit beta OS=Mus musculus GN=Etfb PE=1 SV=3	Q9DCW4	28 kDa	28	11	0
305	Ras GTPase-activating-like protein IQGAP1 OS=Mus musculus GN=Iqgap1 PE=1 SV=2	Q9JKF1	189 kDa	0	45	46
306	Cluster of Proteasome subunit beta type-3 OS=Mus musculus GN=Psmb3 PE=1 SV=1 (Q9R1P1)	Q9R1P1	23 kDa	65	11	92
307	Cluster of Apolipoprotein C-III OS=Mus musculus GN=Apoc3 PE=1 SV=2 (P33622)	P33622	11 kDa	93	45	11
308	Isoform 2 of Acetyl-CoA carboxylase 1 OS=Mus musculus GN=Acaca	Q5SWU9-2	270 kDa	19	0	0
309	Proteasome subunit alpha type-3 OS=Mus musculus GN=Psma3 PE=1 SV=3	O70435	28 kDa	56	56	57
310	Transgelin OS=Mus musculus GN=Tagln PE=1 SV=3	P37804	23 kDa	37	67	34
311	Adenylyl cyclase-associated protein 1 OS=Mus musculus GN=Cap1 PE=1 SV=4	P40124	52 kDa	65	78	34
312	CD5 antigen-like OS=Mus musculus GN=Cd5l PE=1 SV=3	Q9QWK4	39 kDa	1	22	34
313	Chloride intracellular channel protein 1 OS=Mus musculus GN=Clic1 PE=1 SV=3	Q9Z1Q5	27 kDa	37	67	10

314	Disintegrin and metalloproteinase domain-containing protein 21 OS=Mus musculus GN=Adam21 PE=2 SV=1	Q9JI76	81 kDa	37	34	23
315	Adenylosuccinate synthetase isozyme 1 OS=Mus musculus GN=Adssl1 PE=3 SV=1	J3QN31 (+2)	53 kDa	93	12	0
316	Cluster of Isoform 3 of F-actin-capping protein subunit beta OS=Mus musculus GN=Capzb (P47757-4)	P47757-4	34 kDa	56	78	80
317	Guanine nucleotide-binding protein subunit beta-2-like 1 OS=Mus musculus GN=Gnb2l1 PE=1 SV=3	P68040	35 kDa	37	11	11
318	Transaldolase OS=Mus musculus GN=Taldo1 PE=1 SV=2	Q93092	37 kDa	0	56	0
319	Cluster of Ferritin OS=Mus musculus GN=Ftl1 PE=2 SV=1 (Q9CPX4)	Q9CPX4	21 kDa	37	45	18
320	Guanine deaminase OS=Mus musculus GN=Gda PE=1 SV=1	Q9R111	51 kDa	1	34	46
321	Cluster of Protein Gm20431 OS=Mus musculus GN=Gm20431 PE=4 SV=1 (E9PY39)	E9PY39	42 kDa	19	22	34
322	Macrophage colony-stimulating factor 1 receptor OS=Mus musculus GN=Csf1r PE=1 SV=3	P09581	109 kDa	83	56	11
323	Cluster of Dihydropteridine reductase OS=Mus musculus GN=Qdpr PE=1 SV=2 (Q8BVI4)	Q8BVI4	26 kDa	56	45	23
324	Transient receptor potential cation channel subfamily M member 2 OS=Mus musculus GN=Trpm2 PE=2 SV=1	Q5KTC0	172 kDa	0	0	23
325	Cluster of Lysozyme C-2 OS=Mus musculus GN=Lyz2 PE=1 SV=2 (P08905)	P08905	17 kDa	28	56	57
326	Cluster of EGF-containing fibulin-like extracellular matrix protein 1 OS=Mus musculus GN=Efemp1 PE=2 SV=1 (Q8BPB5)	Q8BPB5	55 kDa	0	34	0
327	Cluster of Collagen alpha-1(XV) chain OS=Mus musculus GN=Col15a1 PE=4 SV=1 (A2AJY2)	A2AJY2 [2]	138 kDa	56	45	0
328	78 kDa glucose-regulated protein OS=Mus musculus GN=Hspa5 PE=1 SV=3	P20029	72 kDa	83	20	57
329	14-3-3 protein eta OS=Mus musculus GN=Ywhah PE=1 SV=2	P68510	28 kDa	42	20	33
330	Cluster of Interleukin-1 receptor accessory protein OS=Mus musculus GN=Il1rap PE=2 SV=1 (Q3UVZ1)	Q3UVZ1	79 kDa	37	22	23
331	Cluster of Complement C1r-A subcomponent OS=Mus musculus GN=C1ra PE=1 SV=1 (Q8CG16)	Q8CG16	80 kDa	46	22	0

332	Angiotensin-converting enzyme OS=Mus musculus GN=Ace PE=1 SV=3	P09470	151 kDa	28	22	0
333	Ig kappa chain V-V region MOPC 149 OS=Mus musculus PE=1 SV=1	P01636	12 kDa	83	56	10
334	Cluster of Isoform 2 of Seprase OS=Mus musculus GN=Fap (P97321-2)	P97321-2 [2]	87 kDa	65	45	11
335	Cluster of HMW kininogen-II OS=Mus musculus GN=Kng2 PE=2 SV=1 (Q6S9I3)	Q6S9I3 [2]	71 kDa	74	11	80
336	Cluster of Adenylosuccinate lyase OS=Mus musculus GN=Adsl PE=4 SV=1 (E9Q242)	E9Q242	53 kDa	37	67	11
337	Cluster of Procollagen C-endopeptidase enhancer 1 OS=Mus musculus GN=Pcolce PE=1 SV=2 (Q61398)	Q61398	50 kDa	11	11	11
338	Thioredoxin OS=Mus musculus GN=Txn PE=1 SV=3	P10639	12 kDa	46	16	92
339	Coactosin-like protein OS=Mus musculus GN=Cotl1 PE=1 SV=3	Q9CQI6	16 kDa	0	11	23
340	6-phosphogluconate dehydrogenase, decarboxylating OS=Mus musculus GN=Pgd PE=1 SV=3	Q9DCD0	53 kDa	28	78	80
341	60 kDa heat shock protein, mitochondrial OS=Mus musculus GN=Hspd1 PE=1 SV=1	P63038	61 kDa	19	11	0
342	Cluster of Calmodulin OS=Mus musculus GN=Calm1 PE=1 SV=1 (Q3UKW2)	Q3UKW2	22 kDa	56	34	46
343	Cluster of Talin-2 OS=Mus musculus GN=Tln2 PE=1 SV=1 (E9PUM4)	E9PUM4	272 kDa	19	22	23
344	ATP synthase subunit beta, mitochondrial OS=Mus musculus GN=Atp5b PE=1 SV=2	P56480	56 kDa	1	34	80
345	Cluster of Ig kappa chain V-III region PC 2880/PC 1229 OS=Mus musculus PE=1 SV=1 (P01654)	P01654 [2]	12 kDa	74	78	16
346	Cluster of Chloride intracellular channel protein 4 OS=Mus musculus GN=Clic4 PE=1 SV=3 (Q9QYB1)	Q9QYB1	29 kDa	1	56	80
347	Protein NipSnap homolog 2 OS=Mus musculus GN=Gbas PE=2 SV=1	O55126	33 kDa	93	10	0
348	Peptidyl-prolyl cis-trans isomerase B OS=Mus musculus GN=Ppib PE=1 SV=2	P24369	24 kDa	28	34	46
349	Proteasome subunit alpha type-2 OS=Mus musculus GN=Psma2 PE=1 SV=3	P49722	26 kDa	46	78	69
350	Phosphoglycerate mutase 1 OS=Mus musculus GN=Pgam1 PE=1 SV=3	Q9DBJ1	29 kDa	17	18	14
351	Filamin-B OS=Mus musculus GN=Flnb PE=1 SV=3	Q80X90	278 kDa	10	67	23

352	Cluster of Rab GDP dissociation inhibitor beta OS=Mus musculus GN=Gdi2 PE=1 SV=1 (Q61598)	Q61598	51 kDa	19	90	0
353	Cluster of Collagen alpha-1(XII) chain OS=Mus musculus GN=Col12a1 PE=4 SV=1 (E9PX70)	E9PX70 [3]	334 kDa	0	22	0
354	Cluster of Chitinase-like protein 3 OS=Mus musculus GN=Chil3 PE=1 SV=2 (O35744)	O35744	44 kDa	0	34	13
355	Extracellular superoxide dismutase [Cu-Zn] OS=Mus musculus GN=Sod3 PE=1 SV=1	O09164	27 kDa	37	45	46
356	Isoform Kidney of Band 3 anion transport protein OS=Mus musculus GN=Slc4a1	P04919-2	94 kDa	28	22	11
357	Tripartite motif-containing protein 72 OS=Mus musculus GN=Trim72 PE=1 SV=1	Q1XH17	53 kDa	28	45	0
358	Plastin-2 OS=Mus musculus GN=Lcp1 PE=1 SV=4	Q61233	70 kDa	0	67	92
359	Inositol (Myo)-1(Or 4)-monophosphatase 1 OS=Mus musculus GN=Impa1 PE=2 SV=1	Q924B0	30 kDa	1	45	46
360	Ig heavy chain V region 3-6 OS=Mus musculus GN=Ighv3-6 PE=1 SV=1	P18531	13 kDa	19	11	23
361	Pannexin-2 OS=Mus musculus GN=Panx2 PE=2 SV=2	Q6IMP4	75 kDa	37	11	11
362	Cluster of Tropomyosin beta chain OS=Mus musculus GN=Tpm2 PE=1 SV=1 (P58774)	P58774	33 kDa	1	78	33
363	Cluster of Alpha-amylase 1 OS=Mus musculus GN=Amy1 PE=1 SV=2 (P00687)	P00687	58 kDa	0	22	23
364	Cluster of Selenium-binding protein 1 OS=Mus musculus GN=Selenbp1 PE=1 SV=2 (P17563)	P17563	53 kDa	1	78	11
365	Cluster of Puromycin-sensitive aminopeptidase OS=Mus musculus GN=Npepps PE=1 SV=2 (Q11011)	Q11011	103 kDa	0	78	0
366	Isocitrate dehydrogenase [NAD] subunit alpha, mitochondrial OS=Mus musculus GN=Idh3a PE=1 SV=1	Q9D6R2	40 kDa	37	67	0
367	Cluster of S-formylglutathione hydrolase OS=Mus musculus GN=Esd PE=1 SV=1 (H3BKH6)	H3BKH6 [2]	33 kDa	65	11	34
368	Cluster of 40S ribosomal protein S3 OS=Mus musculus GN=Rps3 PE=1 SV=1 (P62908)	P62908	27 kDa	65	78	11
369	Cluster of Glutathione S-transferase omega-1 OS=Mus musculus GN=Gsto1 PE=2 SV=2 (O09131)	O09131	27 kDa	19	11	0
370	Cluster of Protein disulfide-isomerase A3 OS=Mus musculus GN=Pdia3 PE=1 SV=2 (P27773)	P27773	57 kDa	0	45	34

371	Cluster of 40S ribosomal protein S19 OS=Mus musculus GN=Rps19 PE=1 SV=3 (Q9CZX8)	Q9CZX8	16 kDa	0	11	34
372	Delta-aminolevulinic acid dehydratase OS=Mus musculus GN=Alad PE=1 SV=1	P10518	36 kDa	37	10	92
373	Galectin-1 OS=Mus musculus GN=Lgals1 PE=1 SV=3	P16045	15 kDa	46	34	34
374	Thioredoxin-dependent peroxide reductase, mitochondrial OS=Mus musculus GN=Prdx3 PE=1 SV=1	P20108	28 kDa	37	78	34
375	Serpin B5 OS=Mus musculus GN=Serpinb5 PE=2 SV=1	P70124	42 kDa	0	78	34
376	6-phosphogluconolactonase OS=Mus musculus GN=Pgls PE=2 SV=1	Q9CQ60	27 kDa	19	67	57
377	ES1 protein homolog, mitochondrial OS=Mus musculus GN=D10Jhu81e PE=1 SV=1	Q9D172	28 kDa	1	22	11
378	Cluster of 60S acidic ribosomal protein P0 OS=Mus musculus GN=Rplp0 PE=1 SV=3 (P14869)	P14869	34 kDa	1	90	0
379	Proteasome subunit beta type-1 OS=Mus musculus GN=Psmb1 PE=1 SV=1	O09061	26 kDa	1	56	46
380	Ig kappa chain V-V region L7 (Fragment) OS=Mus musculus GN=Gm10881 PE=1 SV=1	P01642	13 kDa	46	22	57
381	Platelet-activating factor acetylhydrolase OS=Mus musculus GN=Pla2g7 PE=2 SV=2	Q60963	49 kDa	1	22	11
382	Hepatocyte growth factor activator OS=Mus musculus GN=Hgfac PE=1 SV=1	Q9R098	71 kDa	65	22	0
383	Ribonuclease inhibitor OS=Mus musculus GN=Rnh1 PE=1 SV=1	Q91VI7	50 kDa	0	34	0
384	Cluster of Isoform 3 of Elongation factor 1-delta OS=Mus musculus GN=Eef1d (P57776-3)	P57776-3	73 kDa	46	78	23
385	Coagulation factor V OS=Mus musculus GN=F5 PE=1 SV=1	O88783	247 kDa	28	11	23
386	Isoform 2 of Complement factor D OS=Mus musculus GN=Cfd	P03953-2	28 kDa	28	45	46
387	Lactotransferrin OS=Mus musculus GN=Ltf PE=2 SV=4	P08071	78 kDa	0	0	23
388	Myosin-binding protein H OS=Mus musculus GN=Mybph PE=1 SV=2	P70402	53 kDa	74	67	0
389	Succinyl-CoA:3-ketoacid coenzyme A transferase 1, mitochondrial OS=Mus musculus GN=Oxct1 PE=1 SV=1	Q9D0K2	56 kDa	19	34	0
390	26S proteasome non-ATPase regulatory subunit 1 OS=Mus musculus GN=Psmd1 PE=1 SV=1	Q3TXS7	106 kDa	1	22	0

391	Cluster of AP-1 complex subunit beta-1 OS=Mus musculus GN=Ap1b1 PE=1 SV=1 (Q5SVG4)	Q5SVG4 [3]	102 kDa	28	56	11
392	Superoxide dismutase [Mn], mitochondrial OS=Mus musculus GN=Sod2 PE=1 SV=3	P09671	25 kDa	1	45	34
393	40S ribosomal protein SA OS=Mus musculus GN=Rpsa PE=1 SV=4	P14206	33 kDa	65	0	0
394	Serpin H1 OS=Mus musculus GN=Serpinh1 PE=1 SV=3	P19324	47 kDa	0	22	0
395	Actin-related protein 2/3 complex subunit 4 OS=Mus musculus GN=Arpc4 PE=1 SV=3	P59999	20 kDa	28	45	80
396	Carboxymethylenebutenolidase homolog OS=Mus musculus GN=Cmbl PE=2 SV=1	Q8R1G2	28 kDa	28	11	0
397	Adenylate kinase 2, mitochondrial OS=Mus musculus GN=Ak2 PE=1 SV=5	Q9WTP6	26 kDa	37	45	34
398	Decorin OS=Mus musculus GN=Dcn PE=2 SV=1	P28654	40 kDa	1	22	23
399	Cluster of Poly(rC)-binding protein 1 OS=Mus musculus GN=Pcbp1 PE=1 SV=1 (P60335)	P60335 [4]	37 kDa	65	56	0
400	Proteasome subunit beta type-7 OS=Mus musculus GN=Psmb7 PE=1 SV=1	P70195	30 kDa	1	22	34
401	Ubiquitin-like protein ISG15 OS=Mus musculus GN=Isg15 PE=1 SV=4	Q64339	18 kDa	28	90	34
402	Omega-amidase NIT2 OS=Mus musculus GN=Nit2 PE=1 SV=1	Q9JHW2	31 kDa	37	34	23
403	Cluster of MCG1288 OS=Mus musculus GN=Gm15013 PE=3 SV=1 (V9GWY0)	V9GWY0	30 kDa	1	22	0
404	Cluster of Insulin-degrading enzyme OS=Mus musculus GN=Ide PE=1 SV=1 (Q9JHR7)	Q9JHR7	118 kDa	0	67	11
405	Cluster of Sepiapterin reductase OS=Mus musculus GN=Spr PE=1 SV=1 (Q64105)	Q64105	28 kDa	56	0	11
406	Cluster of Isoform 2 of Reticulon-2 OS=Mus musculus GN=Rtn2 (O70622-2)	O70622-2	22 kDa	65	34	0
407	Phosphatidylcholine-sterol acyltransferase OS=Mus musculus GN=Lcat PE=1 SV=2	P16301	50 kDa	56	45	23
408	Tropomyosin alpha-4 chain OS=Mus musculus GN=Tpm4 PE=2 SV=3	Q6IRU2	28 kDa	1	22	46
409	Cystatin E/M OS=Mus musculus GN=Cst6 PE=2 SV=1	Q9D1B1	17 kDa	46	34	23
410	UMP-CMP kinase OS=Mus musculus GN=Cmpk1 PE=1 SV=1	Q9DBP5	22 kDa	0	56	23
411	Proteasome subunit beta type-2 OS=Mus musculus GN=Psmb2 PE=1 SV=1	Q9R1P3	23 kDa	1	11	23

412	Regenerating islet-derived protein 3-beta OS=Mus musculus GN=Reg3b PE=1 SV=1	P35230	19 kDa	0	22	57
413	Collagen alpha-1(IV) chain OS=Mus musculus GN=Col4a1 PE=2 SV=4	P02463	161 kDa	19	11	0
414	Cluster of Leukocyte elastase inhibitor A OS=Mus musculus GN=Serpinb1a PE=1 SV=1 (Q9D154)	Q9D154	43 kDa	1	90	34
415	Cluster of Ig heavy chain V region H8 OS=Mus musculus PE=1 SV=1 (P01788)	P01788 [2]	14 kDa	37	34	11
416	Cluster of Phosphorylase b kinase regulatory subunit beta OS=Mus musculus GN=Phkb PE=1 SV=1 (Q7TSH2)	Q7TSH2	124 kDa	28	11	0
417	Dihydrolipoyl dehydrogenase, mitochondrial OS=Mus musculus GN=Dld PE=1 SV=2	O08749	54 kDa	0	56	11
418	Eukaryotic initiation factor 4A-I OS=Mus musculus GN=Eif4a1 PE=1 SV=1	P60843	46 kDa	19	11	34
419	Ubiquitin-conjugating enzyme E2 N OS=Mus musculus GN=Ube2n PE=1 SV=1	P61089	17 kDa	46	56	80
420	C-1-tetrahydrofolate synthase, cytoplasmic OS=Mus musculus GN=Mthfd1 PE=1 SV=4	Q922D8	101 kDa	1	34	11
421	Low molecular weight phosphotyrosine protein phosphatase OS=Mus musculus GN=Acp1 PE=1 SV=3	Q9D358	18 kDa	74	34	46
422	Chondroitin sulfate proteoglycan 4 OS=Mus musculus GN=Cspg4 PE=1 SV=3	Q8VHY0	252 kDa	0	22	11
423	Isoform 2 of Myosin-11 OS=Mus musculus GN=Myh11	O08638-2	223 kDa	1	22	34
424	Cathepsin B OS=Mus musculus GN=Ctsb PE=1 SV=2	P10605	37 kDa	28	34	69
425	Cytoplasmic aconitate hydratase OS=Mus musculus GN=Aco1 PE=1 SV=3	P28271	98 kDa	19	22	0
426	Transcription elongation factor B polypeptide 2 OS=Mus musculus GN=Tceb2 PE=1 SV=1	P62869	13 kDa	37	0	11
427	Translationally-controlled tumor protein OS=Mus musculus GN=Tpt1 PE=1 SV=1	P63028	19 kDa	56	10	46
428	3-hydroxyisobutyrate dehydrogenase, mitochondrial OS=Mus musculus GN=Hibadh PE=1 SV=1	Q99L13	35 kDa	28	34	23
429	Immunoglobulin J chain OS=Mus musculus GN=Igj PE=2 SV=4	P01592	18 kDa	19	22	34
430	Coagulation factor XIII A chain OS=Mus musculus GN=F13a1 PE=2 SV=3	Q8BH61	83 kDa	28	22	11
431	Cluster of Ras-related protein Rab-5C OS=Mus musculus GN=Rab5c PE=1 SV=2 (P35278)	P35278 [3]	23 kDa	46	34	34

432	Cluster of Serine/threonine-protein phosphatase PP1-gamma catalytic subunit OS=Mus musculus GN=Ppp1cc PE=1 SV=1 (P63087)	P63087	37 kDa	19	34	0
433	Cluster of Phospholipid hydroperoxide glutathione peroxidase, mitochondrial OS=Mus musculus GN=Gpx4 PE=1 SV=4 (O70325)	O70325	22 kDa	1	22	0
434	Uncharacterized protein OS=Mus musculus GN=Gm10260 PE=3 SV=2	F6YVP7	18 kDa	28	56	11
435	Ubiquinone biosynthesis protein COQ9, mitochondrial OS=Mus musculus GN=Coq9 PE=1 SV=1	Q8K1Z0	35 kDa	37	56	0
436	Isochorismatase domain-containing protein 1 OS=Mus musculus GN=Isoc1 PE=1 SV=1	Q91V64	32 kDa	19	22	34
437	Prolyl endopeptidase OS=Mus musculus GN=Prep PE=2 SV=1	Q9QUR6	81 kDa	0	22	0
438	Proteasome subunit beta type-6 OS=Mus musculus GN=Psmb6 PE=1 SV=3	Q60692	25 kDa	28	45	23
439	Ubiquitin-conjugating enzyme E2 L3 OS=Mus musculus GN=Ube2l3 PE=2 SV=1	P68037	18 kDa	28	22	23
440	Cluster of Myosin regulatory light chain 12B OS=Mus musculus GN=Myl12b PE=1 SV=2 (Q3THE2)	Q3THE2	20 kDa	19	34	57
441	Cluster of Very long-chain specific acyl-CoA dehydrogenase, mitochondrial OS=Mus musculus GN=Acadvl PE=1 SV=3 (P50544)	P50544	71 kDa	1	0	0
442	Endoplasmin OS=Mus musculus GN=Hsp90b1 PE=1 SV=2	P08113	92 kDa	1	11	46
443	40S ribosomal protein S15a OS=Mus musculus GN=Rps15a PE=1 SV=2	P62245	15 kDa	19	11	11
444	Enoyl-CoA hydratase, mitochondrial OS=Mus musculus GN=Echs1 PE=1 SV=1	Q8BH95	31 kDa	28	34	11
445	Spectrin beta 1 OS=Mus musculus GN=Sptb PE=1 SV=1	Q3UGX2	268 kDa	1	22	11
446	Cluster of Phosphorylase b kinase gamma catalytic chain, skeletal muscle/heart isoform OS=Mus musculus GN=Phkg1 PE=2 SV=3 (P07934)	P07934	45 kDa	19	0	0
447	Cluster of Ribosomal protein OS=Mus musculus GN=Rpl10a PE=1 SV=1 (Q5XJF6)	Q5XJF6	25 kDa	1	22	0
448	Cluster of Actin-related protein 2/3 complex subunit 3 OS=Mus musculus GN=Arpc3 PE=1 SV=1 (H7BWZ3)	H7BWZ3	20 kDa	0	0	34

449	Cluster of Dehydrogenase/reductase SDR family member 11 OS=Mus musculus GN=Dhrs11 PE=2 SV=1 (Q3U0B3)	Q3U0B3	28 kDa	0	0	23
450	Galectin-3 OS=Mus musculus GN=Lgals3 PE=1 SV=3	P16110 (+1)	28 kDa	1	22	23
451	Cluster of Transforming protein RhoA OS=Mus musculus GN=Rhoa PE=1 SV=1 (Q9QUI0)	Q9QUI0	22 kDa	37	34	23
452	Ig heavy chain V region 914 OS=Mus musculus PE=1 SV=1	P18527	11 kDa	37	34	69
453	Cluster of Isoform 3 of Reticulon-4 OS=Mus musculus GN=Rtn4 (Q99P72-1)	Q99P72-1	22 kDa	28	34	0
454	Cluster of Four and a half LIM domains protein 3 OS=Mus musculus GN=Fhl3 PE=1 SV=2 (Q9R059)	Q9R059	32 kDa	19	22	0
455	Heat shock protein beta-1 OS=Mus musculus GN=Hspb1 PE=1 SV=3	P14602 (+1)	23 kDa	37	11	46
456	Annexin A5 OS=Mus musculus GN=Anxa5 PE=1 SV=1	P48036	36 kDa	56	0	11
457	Importin subunit beta-1 OS=Mus musculus GN=Kpnb1 PE=1 SV=2	P70168	97 kDa	0	34	0
458	Sulfurtransferase OS=Mus musculus GN=Mpst PE=1 SV=1	Q3UW66 (+1)	33 kDa	19	34	0
459	Hydroxyacyl-coenzyme A dehydrogenase, mitochondrial OS=Mus musculus GN=Hadh PE=1 SV=2	Q61425	34 kDa	1	34	0
460	Inorganic pyrophosphatase OS=Mus musculus GN=Ppa1 PE=1 SV=1	Q9D819	33 kDa	1	34	11
461	Calreticulin OS=Mus musculus GN=Calr PE=1 SV=1	P14211	48 kDa	0	22	23
462	AMP deaminase 1 OS=Mus musculus GN=Ampd1 PE=2 SV=2	Q3V1D3	86 kDa	19	22	0
463	Transcobalamin-2 OS=Mus musculus GN=Tcn2 PE=2 SV=1	O88968	48 kDa	1	34	69
464	Protein S100-A8 OS=Mus musculus GN=S100a8 PE=1 SV=3	P27005	10 kDa	0	11	80
465	Thimet oligopeptidase OS=Mus musculus GN=Thop1 PE=1 SV=1	Q8C1A5	78 kDa	0	45	0
466	Cytosolic non-specific dipeptidase OS=Mus musculus GN=Cndp2 PE=1 SV=1	Q9D1A2	53 kDa	0	34	23
467	Glycogen [starch] synthase, muscle OS=Mus musculus GN=Gys1 PE=1 SV=2	Q9Z1E4	84 kDa	1	22	0
468	Ras suppressor protein 1 OS=Mus musculus GN=Rsu1 PE=4 SV=1	A2AUR7 (+1)	30 kDa	1	11	46

469	Cluster of Proteasome activator complex subunit 1 (Fragment) OS=Mus musculus GN=Psme1 PE=4 SV=1 (G3UXZ5)	G3UXZ5	27 kDa	1	22	46
470	Cluster of Annexin A2 OS=Mus musculus GN=Anxa2 PE=1 SV=2 (P07356)	P07356	39 kDa	1	34	46
471	Ubiquinone biosynthesis monooxygenase COQ6 OS=Mus musculus GN=Coq6 PE=1 SV=1	D3YW66	47 kDa	28	45	46
472	Cluster of Protein-L-isoaspartate(D-aspartate) O-methyltransferase OS=Mus musculus GN=Pcmt1 PE=4 SV=1 (E0CYV0)	E0CYV0 [3]	30 kDa	1	34	0
473	Cluster of Catalase OS=Mus musculus GN=Cat PE=1 SV=4 (P24270)	P24270	60 kDa	1	11	34
474	Cluster of PDZ and LIM domain protein 5 OS=Mus musculus GN=Pdlim5 PE=1 SV=4 (Q8CI51)	Q8CI51	63 kDa	28	0	0
475	Long-chain specific acyl-CoA dehydrogenase, mitochondrial OS=Mus musculus GN=Acadl PE=1 SV=2	P51174	48 kDa	1	22	0
476	Inosine triphosphate pyrophosphatase OS=Mus musculus GN=Itpa PE=1 SV=2	Q9D892	22 kDa	19	45	69
477	Ras-related C3 botulinum toxin substrate 1 OS=Mus musculus GN=Rac1 PE=1 SV=1	P63001	21 kDa	46	22	69
478	60S ribosomal protein L7 OS=Mus musculus GN=Rpl7 PE=1 SV=2	P14148	31 kDa	19	11	11
479	Hepatocyte growth factor-like protein OS=Mus musculus GN=Mst1 PE=3 SV=1	E0CXN0	80 kDa	19	11	0
480	Cluster of Glutamate--cysteine ligase regulatory subunit OS=Mus musculus GN=Gclm PE=2 SV=1 (O09172)	O09172	31 kDa	19	22	23
481	Proteasome subunit beta type-8 OS=Mus musculus GN=Psmb8 PE=1 SV=2	P28063	30 kDa	1	0	23
482	Cluster of 26S proteasome non-ATPase regulatory subunit 11 OS=Mus musculus GN=Psmd11 PE=1 SV=3 (Q8BG32)	Q8BG32	47 kDa	19	11	0
483	Keratin, type II cytoskeletal 72 OS=Mus musculus GN=Krt72 PE=3 SV=1	Q6IME9	57 kDa	37	34	0
484	Cluster of Ras-related protein Rap-1A OS=Mus musculus GN=Rap1a PE=2 SV=1 (P62835)	P62835	21 kDa	28	11	0
485	Cluster of Serine/threonine-protein phosphatase 2A 65 kDa regulatory subunit A alpha isoform OS=Mus musculus GN=Ppp2r1a PE=1 SV=3 (Q76MZ3)	Q76MZ3	65 kDa	19	22	0
486	Ras-related protein Rab-11A OS=Mus musculus GN=Rab11a PE=3 SV=1	E9Q3P9	17 kDa	37	11	11

487	Regenerating islet-derived protein 3-gamma OS=Mus musculus GN=Reg3g PE=1 SV=1	O09049	19 kDa	19	34	34
488	26S proteasome non-ATPase regulatory subunit 3 OS=Mus musculus GN=Psmd3 PE=1 SV=3	P14685	61 kDa	0	22	0
489	Cofilin-1 OS=Mus musculus GN=Cfl1 PE=1 SV=3	P18760	19 kDa	83	10	92
490	Cluster of Hydroxyacylglutathione hydrolase, mitochondrial (Fragment) OS=Mus musculus GN=Hagh PE=1 SV=1 (E9PYA3)	E9PYA3	26 kDa	19	45	23
491	Cluster of Dual-specificity protein phosphatase 3 OS=Mus musculus GN=Dusp3 PE=4 SV=1 (B1AQF4)	B1AQF4	23 kDa	83	0	11
492	Cluster of Vascular cell adhesion protein 1 OS=Mus musculus GN=Vcam1 PE=1 SV=1 (P29533)	P29533 [2]	81 kDa	19	0	0
493	Cluster of Thioredoxin domain-containing protein 5 OS=Mus musculus GN=Txndc5 PE=1 SV=2 (Q91W90)	Q91W90	46 kDa	0	22	0
494	Cluster of Actin-related protein 3 OS=Mus musculus GN=Actr3 PE=1 SV=3 (Q99JY9)	Q99JY9	47 kDa	0	34	57
495	Cluster of Obg-like ATPase 1 OS=Mus musculus GN=Ola1 PE=1 SV=1 (Q9CZ30)	Q9CZ30	45 kDa	0	11	0
496	Actin-related protein 2 OS=Mus musculus GN=Actr2 PE=1 SV=1	P61161	45 kDa	0	22	46
497	Dermatopontin OS=Mus musculus GN=Dpt PE=2 SV=1	Q9QZZ6	24 kDa	19	11	23
498	Ig lambda-1 chain C region OS=Mus musculus PE=1 SV=1	P01843	12 kDa	1	56	11
499	SPARC OS=Mus musculus GN=Sparc PE=1 SV=1	P07214 (+1)	34 kDa	0	22	23
500	Ig heavy chain V region MOPC 21 (Fragment) OS=Mus musculus PE=1 SV=1	P01783	15 kDa	37	45	13
501	Destrin OS=Mus musculus GN=Dstn PE=1 SV=3	Q9R0P5	19 kDa	1	22	46
502	Ferritin heavy chain OS=Mus musculus GN=Fth1 PE=1 SV=2	P09528	21 kDa	28	11	23
503	Acyl-coenzyme A thioesterase 13 OS=Mus musculus GN=Acot13 PE=1 SV=1	Q9CQR4	15 kDa	37	22	0
504	Ig kappa chain V-II region 7S34.1 OS=Mus musculus PE=1 SV=1	P01630	12 kDa	37	67	57
505	Cluster of Glycogenin-1 OS=Mus musculus GN=Gyg PE=4 SV=1 (K3W4S6)	K3W4S6 [2]	42 kDa	37	11	0
506	Cluster of 60S ribosomal protein L18a OS=Mus musculus GN=Rpl18a PE=1 SV=1 (P62717)	P62717	21 kDa	0	0	0

507	Ig kappa chain V-V region MOPC 41 OS=Mus musculus GN=Gm5571 PE=1 SV=1	P01639	14 kDa	37	45	34
508	Ig heavy chain V-III region A4 OS=Mus musculus PE=1 SV=1	P01796 (+2)	13 kDa	37	78	46
509	Isoform Smooth muscle of Myosin light polypeptide 6 OS=Mus musculus GN=Myl6	Q60605-2	17 kDa	1	34	13
510	Endoplasmic reticulum resident protein 29 OS=Mus musculus GN=Erp29 PE=1 SV=2	P57759	29 kDa	19	22	23
511	Protein AI182371 OS=Mus musculus GN=AI182371 PE=4 SV=2	A2AS37 (+1)	40 kDa	19	56	34
512	Ras-related protein Rab-7a OS=Mus musculus GN=Rab7a PE=1 SV=2	P51150	23 kDa	28	0	34
513	Cystatin-C OS=Mus musculus GN=Cst3 PE=2 SV=2	P21460	16 kDa	19	11	23
514	Protein disulfide-isomerase A6 OS=Mus musculus GN=Pdia6 PE=1 SV=3	Q922R8	48 kDa	0	22	11
515	Cluster of F-actin-capping protein subunit alpha-2 OS=Mus musculus GN=Capza2 PE=1 SV=3 (P47754)	P47754	33 kDa	1	45	23
516	Cluster of Ribonuclease 4 OS=Mus musculus GN=Rnase4 PE=2 SV=1 (Q9JJH1)	Q9JJH1	17 kDa	1	34	46
517	Cluster of ADP/ATP translocase 1 OS=Mus musculus GN=Slc25a4 PE=1 SV=4 (P48962)	P48962	33 kDa	28	11	0
518	Cluster of Cathepsin D OS=Mus musculus GN=Ctsd PE=1 SV=1 (P18242)	P18242	45 kDa	0	11	0
519	Cluster of MCG130175, isoform CRA_b OS=Mus musculus GN=BC100530 PE=2 SV=1 (Q497J0)	Q497J0	11 kDa	0	11	80
520	Pentraxin-related protein PTX3 OS=Mus musculus GN=Ptx3 PE=1 SV=2	P48759	42 kDa	0	22	34
521	Coagulation factor XIII B chain OS=Mus musculus GN=F13b PE=1 SV=2	Q07968	76 kDa	1	11	0
522	Glutathione S-transferase A4 OS=Mus musculus GN=Gsta4 PE=1 SV=3	P24472	26 kDa	0	22	23
523	Eukaryotic peptide chain release factor subunit 1 OS=Mus musculus GN=Etf1 PE=1 SV=4	Q8BWY3	49 kDa	28	11	0
524	Pyruvate carboxylase OS=Mus musculus GN=Pcx PE=1 SV=1	E9QPD7 (+2)	130 kDa	19	0	0
525	Pyruvate dehydrogenase E1 component subunit beta, mitochondrial OS=Mus musculus GN=Pdhb PE=1 SV=1	Q9D051	39 kDa	0	0	0
526	Cluster of Glucose-6-phosphate 1-dehydrogenase X OS=Mus musculus GN=G6pdx PE=1 SV=3 (Q00612)	Q00612	59 kDa	0	34	11

527	Cluster of Ig kappa chain V-VI region XRPC 44 OS=Mus musculus PE=1 SV=1 (P01675)	P01675 [4]	12 kDa	1	0	0
528	Cluster of Rab GDP dissociation inhibitor alpha OS=Mus musculus GN=Gdi1 PE=1 SV=3 (P50396)	P50396	51 kDa	0	0	0
529	Annexin A1 OS=Mus musculus GN=Anxa1 PE=1 SV=2	P10107	39 kDa	0	11	57
530	Ribose-5-phosphate isomerase OS=Mus musculus GN=Rpia PE=2 SV=2	P47968	32 kDa	19	22	34
531	40S ribosomal protein S13 OS=Mus musculus GN=Rps13 PE=1 SV=2	P62301	17 kDa	0	0	0
532	Peptidyl-prolyl cis-trans isomerase FKBP3 OS=Mus musculus GN=Fkbp3 PE=1 SV=2	Q62446	25 kDa	19	45	11
533	Serpin B6 OS=Mus musculus GN=Serpinb6a PE=1 SV=1	F8WIV2	45 kDa	1	11	23
534	Ubiquitin carboxyl-terminal hydrolase isozyme L3 OS=Mus musculus GN=Uchl3 PE=1 SV=2	Q9JKB1	26 kDa	19	34	11
535	Ras-related C3 botulinum toxin substrate 2 OS=Mus musculus GN=Rac2 PE=2 SV=1	Q05144	21 kDa	28	0	80
536	Carboxypeptidase Q OS=Mus musculus GN=Cpq PE=2 SV=1	Q9WVJ3 (+1)	52 kDa	1	11	0
537	60S ribosomal protein L23 OS=Mus musculus GN=Rpl23 PE=1 SV=1	P62830	15 kDa	0	11	11
538	Isoleucine--tRNA ligase, cytoplasmic OS=Mus musculus GN=Iars PE=2 SV=2	Q8BU30	144 kDa	19	0	0
539	Cluster of Carbonyl reductase [NADPH] 2 OS=Mus musculus GN=Cbr2 PE=1 SV=1 (P08074)	P08074	26 kDa	19	22	23
540	Cluster of Mannosyl-oligosaccharide 1,2-alpha-mannosidase IA OS=Mus musculus GN=Man1a1 PE=1 SV=1 (P45700)	P45700	73 kDa	1	11	0
541	Glyceraldehyde-3-phosphate dehydrogenase, testis-specific OS=Mus musculus GN=Gapdhs PE=2 SV=1	Q64467 (+1)	48 kDa	25	12	23
542	Cluster of Serine/threonine-protein phosphatase 2A 65 kDa regulatory subunit A beta isoform OS=Mus musculus GN=Ppp2r1b PE=4 SV=1 (H3BLE7)	H3BLE7	66 kDa	28	11	0
543	Isoform Short of Heterogeneous nuclear ribonucleoprotein A1 OS=Mus musculus GN=Hnrnpa1	P49312-2	29 kDa	1	45	0
544	40S ribosomal protein S14 OS=Mus musculus GN=Rps14 PE=2 SV=3	P62264	16 kDa	0	0	46

545	Carbonyl reductase [NADPH] 3 OS=Mus musculus GN=Cbr3 PE=2 SV=1	Q8K354	31 kDa	1	0	34
546	Pro-cathepsin H OS=Mus musculus GN=Ctsh PE=2 SV=2	P49935	37 kDa	0	22	23
547	Platelet-activating factor acetylhydrolase IB subunit beta OS=Mus musculus GN=Pafah1b2 PE=1 SV=2	Q61206	26 kDa	19	11	34
548	Biliverdin reductase A OS=Mus musculus GN=Blvra PE=2 SV=1	Q9CY64	34 kDa	0	22	0
549	Eosinophil cationic-type ribonuclease 3 OS=Mus musculus GN=Ear3 PE=3 SV=1	O35290	18 kDa	1	0	11
550	Heterogeneous nuclear ribonucleoproteins A2/B1 OS=Mus musculus GN=Hnrnpa2b1 PE=1 SV=2	O88569	37 kDa	0	0	34
551	Programmed cell death 6-interacting protein OS=Mus musculus GN=Pdcd6ip PE=1 SV=3	Q9WU78	96 kDa	1	22	0
552	Ubiquitin-conjugating enzyme E2 K OS=Mus musculus GN=Ube2k PE=1 SV=3	P61087	22 kDa	37	22	11
553	Ubiquitin thioesterase OTUB1 OS=Mus musculus GN=Otub1 PE=1 SV=2	Q7TQI3	31 kDa	19	11	0
554	N(G),N(G)-dimethylarginine dimethylaminohydrolase 2 OS=Mus musculus GN=Ddah2 PE=1 SV=1	Q99LD8	30 kDa	19	0	11
555	Actin-related protein 2/3 complex subunit 5 OS=Mus musculus GN=Arpc5 PE=2 SV=3	Q9CPW4	16 kDa	19	11	23
556	Cadherin-1 OS=Mus musculus GN=Cdh1 PE=1 SV=1	P09803	98 kDa	0	22	0
557	Cluster of Ig kappa chain V-III region PC 7175 OS=Mus musculus PE=1 SV=1 (P01671)	P01671	12 kDa	46	90	15
558	Thyroxine-binding globulin OS=Mus musculus GN=Serpina7 PE=2 SV=1	P61939	47 kDa	0	22	34
559	Eukaryotic initiation factor 4A-II OS=Mus musculus GN=Eif4a2 PE=3 SV=1	E9Q561	36 kDa	28	11	0
560	Cluster of Retinal dehydrogenase 1 OS=Mus musculus GN=Aldh1a1 PE=1 SV=5 (P24549)	P24549	54 kDa	0	0	0
561	Isoform 2 of Guanidinoacetate N-methyltransferase OS=Mus musculus GN=Gamt	O35969-2	28 kDa	37	0	0
562	Dual specificity phosphatase DUPD1 OS=Mus musculus GN=Dupd1 PE=2 SV=1	Q8BK84	24 kDa	28	11	0
563	EMILIN-1 OS=Mus musculus GN=Emilin1 PE=1 SV=1	Q99K41	108 kDa	19	0	0
564	Cluster of Isoform 2 of Alpha-synuclein OS=Mus musculus GN=Snca (O55042-2)	O55042-2	12 kDa	19	11	34

565	Myosin light chain 3 OS=Mus musculus GN=Myl3 PE=1 SV=4	P09542	22 kDa	0	0	15
566	60S ribosomal protein L30 OS=Mus musculus GN=Rpl30 PE=2 SV=2	P62889	13 kDa	1	11	46
567	60S acidic ribosomal protein P2 OS=Mus musculus GN=Rplp2 PE=1 SV=3	P99027	12 kDa	1	56	23
568	Ubiquitin carboxyl-terminal hydrolase OS=Mus musculus GN=Usp5 PE=1 SV=1	Q3U4W8	93 kDa	0	22	0
569	Phospholipid transfer protein OS=Mus musculus GN=Pltp PE=4 SV=1	A2A5K2	49 kDa	1	0	0
570	Tubulin beta-1 chain OS=Mus musculus GN=Tubb1 PE=1 SV=1	A2AQ07	50 kDa	19	34	11
571	Cathelin-related antimicrobial peptide OS=Mus musculus GN=Camp PE=2 SV=1	P51437	20 kDa	0	22	46
572	Heterogeneous nuclear ribonucleoprotein A/B OS=Mus musculus GN=Hnrnpab PE=1 SV=1	Q20BD0 (+2)	36 kDa	0	22	34
573	AP-2 complex subunit alpha-2 OS=Mus musculus GN=Ap2a2 PE=1 SV=2	P17427	104 kDa	1	22	0
574	Apolipoprotein C-IV OS=Mus musculus GN=Apoc4 PE=2 SV=1	Q61268	14 kDa	37	11	34
575	Epididymal secretory protein E1 OS=Mus musculus GN=Npc2 PE=1 SV=1	Q9Z0J0	16 kDa	0	11	23
576	Cluster of EF-hand domain-containing protein D2 OS=Mus musculus GN=Efhd2 PE=1 SV=1 (Q8C845)	Q8C845	27 kDa	19	0	0
577	Cluster of Coronin-1A OS=Mus musculus GN=Coro1a PE=1 SV=5 (O89053)	O89053	51 kDa	0	11	23
578	Heat shock protein beta-2 OS=Mus musculus GN=Hspb2 PE=2 SV=2	Q99PR8	20 kDa	28	0	0
579	[illegible] phosphoribosyltransferase OS=Mus musculus GN=Hprt1 PE=1 SV=3	P00493	25 kDa	0	34	11
580	Ras-related protein Rab-18 OS=Mus musculus GN=Rab18 PE=2 SV=2	P35293	23 kDa	28	0	0
581	Acylpyruvase FAHD1, mitochondrial OS=Mus musculus GN=Fahd1 PE=1 SV=2	Q8R0F8	25 kDa	0	22	11
582	Cluster of COP9 signalosome complex subunit 7a (Fragment) OS=Mus musculus GN=Cops7a PE=4 SV=2 (D3Z440)	D3Z440 [2]	25 kDa	19	22	0
583	Rho-related GTP-binding protein RhoB OS=Mus musculus GN=Rhob PE=1 SV=1	P62746	22 kDa	1	11	0
584	Cluster of D-dopachrome decarboxylase OS=Mus musculus GN=Ddt PE=1 SV=3 (O35215)	O35215	13 kDa	28	22	11

585	Cluster of ATP synthase subunit O, mitochondrial OS=Mus musculus GN=Atp5o PE=1 SV=1 (Q9DB20)	Q9DB20	23 kDa	19	11	0
586	Cluster of Ras-related protein Rab-2A OS=Mus musculus GN=Rab2a PE=1 SV=1 (P53994)	P53994	24 kDa	19	0	0
587	Cluster of L-selectin OS=Mus musculus GN=Sell PE=2 SV=1 (P18337)	P18337	42 kDa	19	0	0
588	COP9 signalosome complex subunit 8 OS=Mus musculus GN=Cops8 PE=1 SV=1	Q8VBV7	23 kDa	28	22	0
589	Apoptosis-associated speck-like protein containing a CARD OS=Mus musculus GN=Pycard PE=1 SV=1	Q9EPB4	21 kDa	0	0	23
590	Protein MEMO1 OS=Mus musculus GN=Memo1 PE=1 SV=1	Q91VH6	34 kDa	0	22	0
591	Serine/threonine-protein phosphatase 2A catalytic subunit alpha isoform OS=Mus musculus GN=Ppp2ca PE=1 SV=1	P63330	36 kDa	19	22	0
592	Histone H2A OS=Mus musculus GN=H2afz PE=2 SV=1	Q3UA95	11 kDa	28	11	16
593	L-lactate dehydrogenase C chain OS=Mus musculus GN=Ldhc PE=1 SV=2	P00342	36 kDa	26	20	80
594	Cluster of Elongation factor 1-beta OS=Mus musculus GN=Eef1b PE=1 SV=5 (O70251)	O70251	25 kDa	19	22	23
595	Cluster of Isopentenyl-diphosphate Delta-isomerase 1 OS=Mus musculus GN=Idi1 PE=2 SV=1 (P58044)	P58044	26 kDa	0	22	0
596	Cluster of Malectin OS=Mus musculus GN=Mlec PE=2 SV=2 (Q6ZQI3)	Q6ZQI3	32 kDa	0	11	0
597	Alpha-soluble NSF attachment protein OS=Mus musculus GN=Napa PE=1 SV=1	Q9DB05	33 kDa	28	11	0
598	2,4-dienoyl-CoA reductase, mitochondrial OS=Mus musculus GN=Decr1 PE=1 SV=1	Q9CQ62	36 kDa	19	11	0
599	40S ribosomal protein S8 OS=Mus musculus GN=Rps8 PE=1 SV=2	P62242	24 kDa	0	0	23
600	Cluster of Ribose-phosphate pyrophosphokinase 1 OS=Mus musculus GN=Prps1 PE=1 SV=4 (Q9D7G0)	Q9D7G0	35 kDa	0	11	23
601	Ketimine reductase mu-crystallin OS=Mus musculus GN=Crym PE=1 SV=1	O54983	34 kDa	0	67	0
602	NAD(P)H-hydrate epimerase OS=Mus musculus GN=Apoa1bp PE=1 SV=1	Q8K4Z3	31 kDa	19	11	0
603	Biglycan OS=Mus musculus GN=Bgn PE=2 SV=1	P28653	42 kDa	0	0	23

604	SH3 domain-binding glutamic acid-rich-like protein OS=Mus musculus GN=Sh3bgrl PE=3 SV=1	Q9JJU8	13 kDa	0	22	11
605	Vesicle-associated membrane protein-associated protein A OS=Mus musculus GN=Vapa PE=1 SV=2	Q9WV55	28 kDa	1	0	0
606	Cluster of Ig kappa chain V-III region PC 7043 OS=Mus musculus PE=1 SV=1 (P01665)	P01665 [2]	12 kDa	28	45	14
607	Cluster of Capping protein (Actin filament), gelsolin-like OS=Mus musculus GN=Capg PE=1 SV=1 (Q99LB4)	Q99LB4	39 kDa	0	34	11
608	Cluster of Aldehyde dehydrogenase, mitochondrial OS=Mus musculus GN=Aldh2 PE=1 SV=1 (P47738)	P47738	57 kDa	0	34	0
609	Plasminogen activator inhibitor 2, macrophage OS=Mus musculus GN=Serpinb2 PE=2 SV=1	P12388	46 kDa	0	0	23
610	Transcription elongation factor B polypeptide 1 OS=Mus musculus GN=Tceb1 PE=1 SV=1	P83940	12 kDa	19	0	0
611	Ig kappa chain V-I region S107A OS=Mus musculus GN=Igkv7-33 PE=4 SV=1	P01632	13 kDa	56	34	23
612	Vesicle-associated membrane protein, associated protein B and C OS=Mus musculus GN=Vapb PE=2 SV=1	Q8BH80 (+1)	27 kDa	19	0	0
613	Guanine nucleotide-binding protein G(I)/G(S)/G(T) subunit beta-3 OS=Mus musculus GN=Gnb3 PE=1 SV=2	Q61011	37 kDa	0	0	0
614	Serum amyloid A-1 protein OS=Mus musculus GN=Saa1 PE=1 SV=2	P05366	14 kDa	0	0	16
615	60S acidic ribosomal protein P1 OS=Mus musculus GN=Rplp1 PE=2 SV=1	P47955	11 kDa	0	45	23
616	GTP-binding protein SAR1b OS=Mus musculus GN=Sar1b PE=1 SV=1	Q9CQC9	22 kDa	46	11	0
617	Eukaryotic translation initiation factor 3 subunit L OS=Mus musculus GN=Eif3l PE=1 SV=1	Q8QZY1	67 kDa	0	22	0
618	Serine/threonine-protein phosphatase 2A 55 kDa regulatory subunit B alpha isoform OS=Mus musculus GN=Ppp2r2a PE=1 SV=1	Q6P1F6	52 kDa	1	34	0
619	ATP-dependent 6-phosphofructokinase, liver type OS=Mus musculus GN=Pfkl PE=1 SV=4	P12382	85 kDa	0	0	0
620	Myosin light chain 4 OS=Mus musculus GN=Myl4 PE=2 SV=3	P09541	21 kDa	0	0	0
621	Ig heavy chain V region 3 OS=Mus musculus GN=Ighv1-61 PE=1 SV=1	P01749	13 kDa	28	0	11

622	Cluster of Peroxiredoxin-4 (Fragment) OS=Mus musculus GN=Prdx4 PE=4 SV=1 (B1AZS9)	B1AZS9	26 kDa	65	67	46
623	Cluster of Prefoldin subunit 2 OS=Mus musculus GN=Pfdn2 PE=2 SV=2 (O70591)	O70591	17 kDa	19	34	0
624	F-actin-capping protein subunit alpha-1 OS=Mus musculus GN=Capza1 PE=1 SV=4	P47753 (+1)	33 kDa	0	22	11
625	Ig kappa chain V-III region PC 3741/TEPC 111 OS=Mus musculus PE=1 SV=1	P01660	12 kDa	46	45	10
626	PRA1 family protein 3 OS=Mus musculus GN=Arl6ip5 PE=1 SV=2	Q8R5J9	22 kDa	0	0	0
627	GTP-binding protein SAR1a OS=Mus musculus GN=Sar1a PE=2 SV=1	Q99JZ4	22 kDa	37	11	11
628	Ribonuclease UK114 OS=Mus musculus GN=Hrsp12 PE=1 SV=3	P52760	14 kDa	1	11	0
629	Ig kappa chain V-III region ABPC 22/PC 9245 OS=Mus musculus PE=1 SV=1	P01662 (+1)	12 kDa	19	34	69
630	Cluster of Glia maturation factor gamma OS=Mus musculus GN=Gmfg PE=1 SV=1 (Q9ERL7)	Q9ERL7	17 kDa	0	0	34
631	Tubulin polymerization-promoting protein family member 3 OS=Mus musculus GN=Tppp3 PE=1 SV=1	Q9CRB6	19 kDa	1	0	23
632	Cluster of Heme oxygenase 1 OS=Mus musculus GN=Hmox1 PE=1 SV=1 (P14901)	P14901	33 kDa	0	0	23
633	Diphosphoinositol polyphosphate phosphohydrolase 1 OS=Mus musculus GN=Nudt3 PE=3 SV=1	H3BLR8 (+1)	14 kDa	19	0	0
634	H-2 class I histocompatibility antigen, D-K alpha chain OS=Mus musculus GN=H2-D1 PE=1 SV=1	P14426	41 kDa	19	22	11
635	Heterogeneous nuclear ribonucleoprotein D0 (Fragment) OS=Mus musculus GN=Hnrnpd PE=1 SV=1	E9Q5B6	12 kDa	0	22	11
636	Astrocytic phosphoprotein PEA-15 OS=Mus musculus GN=Pea15 PE=1 SV=1	Q62048 (+1)	15 kDa	0	0	23
637	Calmodulin-4 OS=Mus musculus GN=Calm4 PE=2 SV=2	Q9JM83	17 kDa	0	0	23
638	Protein FAM49B OS=Mus musculus GN=Fam49b PE=2 SV=1	Q921M7	37 kDa	19	0	11
639	Ig lambda-1 chain V region OS=Mus musculus PE=1 SV=2	P01723 (+1)	12 kDa	19	11	0
