# Supplementary material for: Muscle Tissue Damage Induced by the Venom of Bothrops asper: Identification of Early and Late Pathological Events through Proteomic Analysis
Source: PLoS Negl Trop Dis. 2016 Apr 1;10(4):e0004599. doi: 10.1371/journal.pntd.0004599 (PMC4818029; doi:10.1371/journal.pntd.0004599)
Supplement: S2 Table — (PDF) [file pntd.0004599.s002.pdf]

S2 Table. Intracellular proteins identified in wound exudates collected from mice at 1, 6 and 24 h after injection of *B. asper* venom.

Protein	Accession Number	Molecular mass	Quantitative Value		
			1h	6h	24h
**Proteins which changed at least three-fold at one time as compared to another time**					
Creatine kinase M-type	P07310	43 kDa	651	404	96
Fructose-bisphosphate aldolase	A6ZI44	45 kDa	357	216	109
Phosphorylase	E9PUM3	88 kDa	300	294	47
Carboxylesterase 1C	P23953	61 kDa	200	168	0
Alpha-actinin-2	Q9JI91	104 kDa	190	298	59
Alpha-actinin-3	O88990	103 kDa	161	206	55
Actin, alpha skeletal muscle	P68134	42 kDa	120	258	220
L-lactate dehydrogenase	G5E8N5	40 kDa	105	87	34
Triosephosphate isomerase	P17751	32 kDa	98	96	27
Bisphosphoglycerate mutase	O70250	29 kDa	93	15	14
Cofilin-1	P18760	19 kDa	83	10	92
Glutathione peroxidase 1	P11352	22kDa	83	13	14
Isoform 2 of Myc box-dependent-interacting protein 1	O08539-2 [2]	48 kDa	83	22	0
Peroxiredoxin-5, mitochondrial	P99029 [2]	22 kDa	83	16	17
Sarcoplasmic/endoplasmic reticulum calcium ATPase 1	Q8R429 [2]	109 kDa	80	77	0
Flavin reductase (NADPH)	Q923D2	22 kDa	74	15	22
Myosin-binding protein H	P70402	53 kDa	74	67	0
Ubiquitin-40S ribosomal protein S27a	P62983 [2]	18 kDa	74	15	26
UTP--glucose-1-phosphate uridylyltransferase	Q91ZJ5	57 kDa	74	78	11
ATP-dependent 6-phosphofructokinase, muscle type	P47857	85 kDa	68	27	0
L-lactate dehydrogenase B chain	P16125	37 kDa	65	18	80
Malate dehydrogenase, mitochondrial	P08249	36 kDa	65	63	14
Myosin-9	Q8VDD5	226 kDa	65	10	31
Phosphoglycerate kinase 1	P09411	45 kDa	58	47	15
Annexin A5	P48036	36 kDa	56	0	11
Citrate synthase, mitochondrial	Q9CZU6 [2]	52 kDa	56	17	11
Sepiapterin reductase	Q64105	28 kDa	56	0	11
Heat shock cognate 71 kDa protein	P63017 [4]	71 kDa	51	73	17
Aconitate hydratase, mitochondrial	Q99KI0	85 kDa	48	99	10
GTP-binding protein SAR1b	Q9CQC9	22 kDa	46	11	0
Isoform 3 of Elongation factor 1-delta	P57776-3	73 kDa	46	78	23
Isoform Cytoplasmic of Fumarate hydratase, mitochondrial	P97807-2	50 kDa	46	15	69
Thioredoxin	P10639	12 kDa	46	16	92
Myosin-4	Q5SX39 [9]	223 kDa	43	620	529
14-3-3 protein eta	P68510	28 kDa	42	0	33
[illegible]	[illegible]	[illegible]	[illegible]	[illegible]	[illegible]

Guanine nucleotide-binding protein subunit beta-2-like 1	P68040	35 kDa	37	11	11
Heat shock protein beta-1	P14602 (+1)	23 kDa	37	11	46
Isocitrate dehydrogenase [NAD] subunit alpha, mitochondrial	Q9D6R2	40 kDa	37	67	0
Isoform 2 of Guanidinoacetate N-methyltransferase	O35969-2	28 kDa	37	0	0
Transcription elongation factor B polypeptide 2	P62869	13 kDa	37	0	11
Ubiquinone biosynthesis protein COQ9, mitochondrial	Q8K1Z0	35 kDa	37	56	0
Ubiquitin-conjugating enzyme E2 K	P61087	22 kDa	37	22	11
Glucose-6-phosphate isomerase	P06745	63 kDa	31	52	17
3-ketoacyl-CoA thiolase, mitochondrial	Q8BWT1	42 kDa	28	90	11
Electron transfer flavoprotein subunit beta	Q9DCW4	28 kDa	28	11	0
Eukaryotic initiation factor 4A-II	E9Q561	36 kDa	28	11	0
Heat shock protein beta-2	Q99PR8	20 kDa	28	0	0
Phosphorylase b kinase regulatory subunit beta	Q7TSH2	124 kDa	28	11	0
Protein disulfide-isomerase	P09103	57 kDa	28	17	69
Superoxide dismutase [Cu-Zn]	P08228	16 kDa	28	10	57
Ubiquitin-like protein ISG15	Q64339	18 kDa	28	90	34
L-lactate dehydrogenase C chain	P00342	36 kDa	26	20	80
Glyceraldehyde-3-phosphate dehydrogenase, testis-specific	Q64467 (+1)	48 kDa	25	12	23
Transitional endoplasmic reticulum ATPase	Q01853	89 kDa	25	35	15
Carboxypeptidase N subunit 2	Q9DBB9	60 kDa	23	41	11
Peptidyl-prolyl cis-trans isomerase A	P17742	18 kDa	22	67	24
Adenylate kinase isoenzyme 1	Q9R0Y5 [2]	22 kDa	20	78	23
2,4-dienoyl-CoA reductase, mitochondrial	Q9CQ62	36 kDa	19	11	0
26S proteasome non-ATPase regulatory subunit 11	Q8BG32	47 kDa	19	11	0
60 kDa heat shock protein, mitochondrial	P63038	61 kDa	19	11	0
AMP deaminase 1	Q3V1D3	86 kDa	19	22	0
ATP synthase subunit O, mitochondrial	Q9DB20	23 kDa	19	11	0
Cytoplasmic aconitate hydratase	P28271	98 kDa	19	22	0
Eukaryotic initiation factor 4A-I	P60843	46 kDa	19	11	34
Glutathione S-transferase omega-1	O09131	27 kDa	19	11	0
Isocitrate dehydrogenase [NADP], mitochondrial	P54071	51 kDa	19	78	0
Isoleucine--tRNA ligase, cytoplasmic	Q8BU30	144 kDa	19	0	0
Myosin regulatory light chain 12B	Q3THE2	20 kDa	19	34	57

Peptidyl-prolyl cis-trans isomerase FKBP3	Q62446	25 kDa	19	45	11
Phosphorylase b kinase gamma catalytic chain	P07934	45 kDa	19	0	0
Succinyl-CoA:3-ketoacid coenzyme A transferase 1, mitochondrial	Q9D0K2	56 kDa	19	34	0
Sulfurtransferase	Q3UW66 (+1)	33 kDa	19	34	0
Transcription elongation factor B polypeptide 1	P83940	12 kDa	19	0	0
Ubiquitin carboxyl-terminal hydrolase isozyme L3	Q9JKB1	26 kDa	19	34	11
Ubiquitin thioesterase OTUB1	Q7TQI3	31 kDa	19	11	0
Clathrin heavy chain 1	Q68FD5	192 kDa	17	18	92
Cofilin-2	P45591	19 kDa	17	78	46
Elongation factor 1-gamma	Q9D8N0	50 kDa	16	22	69
Glutathione S-transferase P 1	P19157	24 kDa	16	15	80
Fatty acid-binding protein, heart	P11404	15 kDa	15	78	0
Electron transfer flavoprotein subunit alpha, mitochondrial	Q99LC5	35 kDa	14	78	0
Phosphatidylethanolamine-binding protein 1	P70296	21 kDa	14	17	57
Carboxypeptidase N catalytic chain	Q9JJN5	52 kDa	12	90	92
Cytoplasmic dynein 1 heavy chain 1	Q9JHU4	532 kDa	12	45	11
Ubiquitin-like modifier-activating enzyme 1	Q02053	118 kDa	12	19	46
Elongation factor 2	P58252	95 kDa	11	36	46
GTP-binding nuclear protein Ran, testis-specific isoform	Q61820 [2]	24 kDa	11	34	80
Myosin light chain 1/3, skeletal muscle isoform	P05977 [2]	21 kDa	10	26	59
Protein S100-A9	P31725	13 kDa	1	56	22
26S proteasome non-ATPase regulatory subunit 1	Q3TXS7	106 kDa	1	22	0
Alcohol dehydrogenase class-3	P28474	40 kDa	1	34	11
Annexin A2	P07356	39 kDa	1	34	46
ATP synthase subunit beta, mitochondrial	P56480	56 kDa	1	34	80
C-1-tetrahydrofolate synthase, cytoplasmic	Q922D8	101 kDa	1	34	11
Carbonyl reductase [NADPH] 3	Q8K354	31 kDa	1	0	34
Carboxypeptidase Q	Q9WVJ3 (+1)	52 kDa	1	11	0
Catalase	P24270	60 kDa	1	11	34
Destrin	Q9R0P5	19 kDa	1	22	46
Endoplasmin	P08113	92 kDa	1	11	46
ES1 protein homolog, mitochondrial	Q9D172	28 kDa	1	22	11
F-actin-capping protein subunit alpha-2	P47754	33 kDa	1	45	23
Guanine deaminase	Q9R111	51 kDa	1	34	46

Hydroxyacyl-coenzyme A dehydrogenase, mitochondrial	Q61425	34 kDa	1	34	0
Isoform 3 of 2-oxoglutarate dehydrogenase, mitochondrial	Q60597-3	118 kDa	1	34	0
Long-chain specific acyl-CoA dehydrogenase, mitochondrial	P51174	48 kDa	1	22	0
Myosin-10	Q3UH59	233 kDa	1	34	23
Phospholipid hydroperoxide glutathione peroxidase, mitochondrial	O70325	22 kDa	1	22	0
Phospholipid transfer protein	A2A5K2	49 kDa	1	0	0
Protein-L-isoaspartate(D-aspartate) O-methyltransferase	E0CYV0 [3]	30 kDa	1	34	0
Rho-related GTP-binding protein RhoB	P62746	22 kDa	1	11	0
Succinate dehydrogenase [ubiquinone] iron-sulfur subunit, mitochondrial	Q9CQA3	32 kDa	1	78	92
Superoxide dismutase [Mn], mitochondrial	P09671	25 kDa	1	45	34
Tropomyosin alpha-3 chain	E9Q5J9 [5]	33 kDa	1	67	23
Tropomyosin alpha-4 chain	Q6IRU2	28 kDa	1	22	46
Tropomyosin beta chain	P58774	33 kDa	1	78	33
Very long-chain specific acyl-CoA dehydrogenase, mitochondrial	P50544	71 kDa	1	0	0
Xanthine dehydrogenase/oxidase	Q00519	147 kDa	1	22	34
Protein S100-A8	P27005	10 kDa	0	11	80
Isoform Smooth muscle of Myosin light polypeptide 6	Q60605-2	17 kDa	0	34	13
26S proteasome non-ATPase regulatory subunit 3	P14685	61 kDa	0	22	0
Actin-related protein 2	P61161	45 kDa	0	22	46
Actin-related protein 2/3 complex subunit 3	H7BWZ3	20 kDa	0	0	34
Actin-related protein 3	Q99JY9	47 kDa	0	34	57
Acylpyruvase FAHD1, mitochondrial	Q8R0F8	25 kDa	0	22	11
Aldehyde dehydrogenase, mitochondrial	P47738	57 kDa	0	34	0
Annexin A1	P10107	39 kDa	0	11	57
Biliverdin reductase A	Q9CY64	34 kDa	0	22	0
Calmodulin-4	Q9JM83	17 kDa	0	0	23
Calreticulin	P14211	48 kDa	0	22	23
Capping protein (Actin filament), gelsolin-like	Q99LB4	39 kDa	0	34	11
Cathepsin D	P18242	45 kDa	0	11	0
Cytosolic non-specific dipeptidase	Q9D1A2	53 kDa	0	34	23
Dehydrogenase/reductase SDR family member 11	Q3U0B3	28 kDa	0	0	23
Dihydrolipoyl dehydrogenase, mitochondrial	O08749	54 kDa	0	56	11

Eukaryotic translation initiation factor 3 subunit L	Q8QZY1	67 kDa	0	22	0
F-actin-capping protein subunit alpha-1	P47753 (+1)	33 kDa	0	22	11
Glucose-6-phosphate 1-dehydrogenase X	Q00612	59 kDa	0	34	11
Glutathione S-transferase A4	P24472	26 kDa	0	22	23
Heme oxygenase 1	P14901	33 kDa	0	0	23
Hypoxanthine-guanine phosphoribosyltransferase	P00493	25 kDa	0	34	11
Isopentenyl-diphosphate Delta-isomerase 1	P58044	26 kDa	0	22	0
Ketimine reductase mu-crystallin	O54983	34 kDa	0	67	0
Myosin light chain 3	P09542	22 kDa	0	0	15
Obg-like ATPase 1	Q9CZ30	45 kDa	0	11	0
Protein disulfide-isomerase A3	P27773	57 kDa	0	45	34
Protein disulfide-isomerase A6	Q922R8	48 kDa	0	22	11
Rho GDP-dissociation inhibitor 2	Q61599	23 kDa	0	10	11
Ribose-phosphate pyrophosphokinase 1	Q9D7G0	35 kDa	0	11	23
Succinyl-CoA ligase [ADP-forming] subunit beta, mitochondrial	Q9Z2I9	50 kDa	0	45	0
5	Q91W90	46 kDa	0	22	0
Ubiquitin carboxyl-terminal hydrolase	Q9JKB1	26 kDa	0	22	0
UMP-CMP kinase	Q9DBP5	22 kDa	0	56	23
**Proteins which did not change more than three-fold at any time as compared to another time**					
Hemoglobin subunit beta-2	P02089	16 kDa	746	924	1,330
Beta-enolase	P21550	47 kDa	216	147	73
Isoform M1 of Pyruvate kinase PKM	P52280-2	58k Da	195	169	81
Vitamin D-binding protein	P21614	54 kDa	178	251	134
Myosin-binding protein C, fast-type	Q5XKE0	127 kDa	121	108	46
Fatty acid synthase	P19096	272 kDa	84	73	32
Gelsolin	P13020	86 kDa	76	76	92
Peptidyl-prolyl cis-trans isomerase C	P30412	23 kDa	74	90	92
Phosphoglycerate mutase 2	O70250	29 kDa	69	98	34
Phosphoglucomutase-1	Q9D0F9 [2]	61 kDa	68	34	69
Peroxiredoxin-4 (Fragment)	B1AZS9	26 kDa	65	67	46
Inhibitor of carbonic anhydrase	Q9DBD0	77 kDa	58	64	62
Parvalbumin alpha	P32848	12 kDa	58	38	37
Aldose reductase	P45376	36 kDa	56	34	23
Calmodulin	Q3UKW2	22 kDa	56	34	46
Carbonic anhydrase 3	P16015	29 kDa	56	53	25
Dihydropteridine reductase	Q8BVI4	26 kDa	56	45	23
Enoyl-CoA delta isomerase 1, mitochondrial	P42125	32 kDa	56	56	46
Isoform 3 of F-actin-capping protein subunit beta	P47757-4	34 kDa	56	78	80
Phosphatidylcholine-sterol acyltransferase	P16301	50 kDa	56	45	23

14-3-3 protein gamma	P61982	28 kDa	50	22	33
Glycerol-3-phosphate dehydrogenase [NAD(+)], cytoplasmic	P13707	38 kDa	48	24	34
Eukaryotic translation initiation factor 5A-1	P63242 [2]	17 kDa	46	22	46
Isoform Short of 14-3-3 protein beta/alpha	Q9CQV8-2 [2]	28 kDa	46	52	48
Myosin regulatory light chain 2, skeletal muscle isoform	P97457	19 kDa	46	30	74
Ubiquitin-conjugating enzyme E2 N	P61089	17 kDa	46	56	80
Elongation factor 1-alpha 2	P62631	50 kDa	43	31	23
Malate dehydrogenase, cytoplasmic	P14152	37 kDa	43	34	80
Peroxiredoxin-2	Q61171	22 kDa	43	38	62
14-3-3 protein epsilon	P62259	29 kDa	42	44	33
Heat shock protein HSP 90-beta	P11499 [2]	83 kDa	42	71	26
Retinol-binding protein 4 O	H7BWY6	28 kDa	39	33	31
Carbonic anhydrase 1	P13634	28 kDa	38	19	22
14-3-3 protein sigma	O70456	28 kDa	37	28	35
Adenylate kinase 2, mitochondrial	Q9WTP6	26 kDa	37	45	34
Thioredoxin-dependent peroxide reductase, mitochondrial	P20108	28 kDa	37	78	34
Elongation factor 1-alpha 1	P10126	50 kDa	31	36	29
Glutathione peroxidase 3	P46412	25 kDa	30	22	27
Desmin	P31001 [2]	53 kDa	29	40	27
3-hydroxyisobutyrate dehydrogenase, mitochondrial	Q99L13	35 kDa	28	34	23
6-phosphogluconate dehydrogenase, decarboxylating	Q9DCD0	53 kDa	28	78	80
Actin-related protein 2/3 complex subunit 4	P59999	20 kDa	28	45	80
Cathepsin B	P10605	37 kDa	28	34	69
Enoyl-CoA hydratase, mitochondrial	Q8BH95	31 kDa	28	34	11
Peptidyl-prolyl cis-trans isomerase B	P24369	24 kDa	28	34	46
Ubiquinone biosynthesis monooxygenase COQ6	D3YW66	47 kDa	28	45	46
Ubiquitin-conjugating enzyme E2 L3	P68037	18 kDa	28	22	23
Peroxiredoxin-1	P35700	22 kDa	27	27	19
Aspartate aminotransferase, mitochondrial	P05202	47 kDa	26	25	46
Fatty acid-binding protein, adipocyte	P04117	15 kDa	24	19	19
Maltase-glucoamylase	B5THE2	209 kDa	24	17	14
14-3-3 protein zeta/delta	P63101	28 kDa	20	41	38
Actin-related protein 2/3 complex subunit 5	Q9CPW4	16 kDa	19	11	23
Carbonyl reductase [NADPH] 2	P08074	26 kDa	19	22	23
Elongation factor 1-beta	O70251	25 kDa	19	22	23
Endoplasmic reticulum resident protein [illegible]					

Fatty acid-binding protein, epidermal	Q05816	15 kDa	19	19	15
Glutamate--cysteine ligase regulatory subunit	O09172	31 kDa	19	22	23
Hydroxyacylglutathione hydrolase, mitochondrial	E9PYA3	26 kDa	19	45	23
Isocitrate dehydrogenase [NADP] cytoplasmic	O88844	47 kDa	19	10	23
Ribose-5-phosphate isomerase	P47968	32 kDa	19	22	34
Creatine kinase S-type, mitochondrial	Q6P8J7	47 kDa	17	28	11
Phosphoglycerate mutase 1	Q9DBJ1	29 kDa	17	18	14
Clusterin	Q06890	52 kDa	16	16	17
Nucleoside diphosphate kinase	E9PZF0 [2]	30 kDa	16	19	18
Rho GDP-dissociation inhibitor 1	Q99PT1	23 kDa	14	11	11
Glutathione S-transferase Mu 2	P15626 [3]	26 kDa	13	22	13
Aspartate aminotransferase, cytoplasmic	P05201	46 kDa	11	18	11
