# Supplementary material for: Muscle Tissue Damage Induced by the Venom of Bothrops asper: Identification of Early and Late Pathological Events through Proteomic Analysis
Source: PLoS Negl Trop Dis. 2016 Apr 1;10(4):e0004599. doi: 10.1371/journal.pntd.0004599 (PMC4818029; doi:10.1371/journal.pntd.0004599)
Supplement: S3 Table — (PDF) [file pntd.0004599.s003.pdf]

S3 Table. Membrane proteins identified in wound exudates collected from mice at 1, 6 and 24 h after injection of *B. asper* venom.

Protein	Accession Number	Molecular Weight	Quantitative Value		
			1 h	6 h	24 h
**Proteins which changed at least three-fold at one time as compared to another time**					
Macrophage colony-stimulating factor 1 receptor	P09581	109 kDa	83	56	11
Angiotensin-converting enzyme	P09470	151 kDa	28	22	0
Ryanodine receptor 1	E9PZQ0	565 kDa	19	45	0
Epidermal growth factor receptor	Q01279	135 kDa	17	24	92
Cadherin-1	P09803	98 kDa	0	22	0
Prolow-density lipoprotein receptor-related protein 1	Q91ZX7	505 kDa	0	34	46
Transient receptor potential cation channel subfamily M member 2	Q5KTC0	172 kDa	0	0	23
**Proteins which did not change more than three-fold at any time as compared to another time**					
ATP-binding cassette sub-family A member 8-B	Q8K440	183 kDa	93	45	57
Interleukin-1 receptor accessory protein	Q3UVZ1	79 kDa	37	22	23
Leukemia inhibitory factor receptor	P42703 [2]	123 kDa	33	25	42
H-2 class I histocompatibility antigen, D-K alpha chain	P14426	41 kDa	19	22	11
H-2 class I histocompatibility antigen, Q10 alpha chain	P01898 [5]	37 kDa	18	24	14
Transferrin receptor protein 1	Q62351	86 kDa	12	15	23
