# Supplementary material for: Muscle Tissue Damage Induced by the Venom of Bothrops asper: Identification of Early and Late Pathological Events through Proteomic Analysis
Source: PLoS Negl Trop Dis. 2016 Apr 1;10(4):e0004599. doi: 10.1371/journal.pntd.0004599 (PMC4818029; doi:10.1371/journal.pntd.0004599)
Supplement: S4 Table — (PDF) [file pntd.0004599.s004.pdf]

S4 Table. Coagulation factors identified in wound exudates collected from mice at 1, 6 and 24 h after injection of *B. asper* venom.

Protein	Accession Number	Molecular Mass	Quantitative Value		
			1 h	6 h	24 h
**Proteins which changed at least three-fold at one time as compared to another time**					
Coagulation factor XII	Q80YC5	66 kDa	65	22	69
Coagulation factor X	O88947	54 kDa	28	0	11
Fibrinogen beta chain	Q8K0E8	55 kDa	13	66	108
Coagulation factor XIII B chain	Q07968	76 kDa	1	11	0
**Proteins which did not change more than three-fold at any time as compared to another time**					
C4b-binding protein	P08607	52 kDa	65	34	23
von Willebrand factor (Fragment)	S4R195 [2]	42 kDa	65	90	46
Prothrombin	P19221	70 kDa	54	19	21
Fibrinogen gamma chain	Q8VCM7	49 kDa	50	118	145
Coagulation factor V	O88783	247 kDa	28	11	23
Coagulation factor XIII A chain	Q8BH61	83 kDa	28	22	11
