# Supplementary material for: Muscle Tissue Damage Induced by the Venom of Bothrops asper: Identification of Early and Late Pathological Events through Proteomic Analysis
Source: PLoS Negl Trop Dis. 2016 Apr 1;10(4):e0004599. doi: 10.1371/journal.pntd.0004599 (PMC4818029; doi:10.1371/journal.pntd.0004599)
Supplement: S5 Table — (PDF) [file pntd.0004599.s005.pdf]

S5 Table. Serum proteinase inhibitors identified in wound exudates collected from mice at 1, 6 and 24 h after injection of *B. asper* venom.

Protein	Accession Number	Molecular Weight	Quantitative Value		
			1 h	6 h	24 h
**Proteins which changed at least three-fold at one time as compared to another time**					
Inter alpha-trypsin inhibitor, heavy chain 4	A6X935 [2]	105 kDa	101	82	301
Inter-alpha-trypsin inhibitor heavy chain H3	Q61704	99 kDa	10	13	47
**Proteins which did not change more than three-fold at any time as compared to another time**					
Serine protease inhibitor A3K	P07759 [4]	47 kDa	821	711	1,292
Alpha-2-macroglobulin	D3YW52 [2]	167 kDa	782	527	712
Murinoglobulin-1	P28665	165 kDa	524	360	432
Alpha-1-antitrypsin 1-2	P22599 [4]	46 kDa	461	488	694
Murinoglobulin-2	P28666	162 kDa	274	214	183
Antithrombin-III	P32261	52 kDa	55	70	57
Inter-alpha-trypsin inhibitor heavy chain H1	Q61702	101 kDa	50	49	48
Alpha-2-antiplasmin	Q61247	55 kDa	34	26	15
Inter-alpha trypsin inhibitor, heavy chain 2	G3X977 (+1)	106 kDa	32	46	57
Plasma protease C1 inhibitor	P97290	56 kDa	31	35	34
Alpha-2-macroglobulin-P	Q6GQT1	164 kDa	26	34	57
Fetuin-B	Q9QXC1	43 kDa	18	24	11
